# Two DNA Methyltransferases for Site-Specific 6mA and 5mC DNA Modification in *Xanthomonas euvesicatoria*

**DOI:** 10.3389/fpls.2021.621466

**Published:** 2021-03-24

**Authors:** Hye-Jee Park, Hoon Je Seong, Jongchan Lee, Lynn Heo, Woo Jun Sul, Sang-Wook Han

**Affiliations:** ^1^Department of Plant Science and Technology, Chung-Ang University, Anseong, South Korea; ^2^R and D Innovation Center, Seoul Clinical Laboratories, Yongin, South Korea; ^3^Department of Systems Biotechnology, Chung-Ang University, Anseong, South Korea

**Keywords:** phytopathogen, *Xanthomonas euvesicatoria*, DNA methyltransferase, comparative proteomics, SMRT (single molecule real-time sequencing), virulence

## Abstract

*Xanthomonas euvesicatoria* (*Xe*) is a gram-negative phytopathogenic bacterium that causes bacterial spot disease in tomato/pepper leading to economic losses in plantations. DNA methyltransferases (MTases) are critical for the survival of prokaryotes; however, their functions in phytopathogenic bacteria remain unclear. In this study, we characterized the functions of two putative DNA MTases, XvDMT1 and XvDMT2, in *Xe* by generating XvDMT1- and XvDMT2-overexpressing strains, *Xe*(XvDMT1) and *Xe*(XvDMT2), respectively. Virulence of *Xe*(XvDMT2), but not *Xe*(XvDMT1), on tomato was dramatically reduced. To postulate the biological processes involving XvDMTs, we performed a label-free shotgun comparative proteomic analysis, and results suggest that XvDMT1 and XvDMT2 have distinct roles in *Xe*. We further characterized the functions of XvDMTs using diverse phenotypic assays. Notably, both *Xe*(XvDMT1) and *Xe*(XvDMT2) showed growth retardation in the presence of sucrose and fructose as the sole carbon source, with *Xe*(XvDMT2) being the most severely affected. In addition, biofilm formation and production of exopolysaccharides were declined in *Xe*(XvDMT2), but not *Xe*(XvDMT1). *Xe*(XvDMT2) was more tolerant to EtOH than *Xe*(XvDMT1), which had enhanced tolerance to sorbitol but decreased tolerance to polymyxin B. Using single-molecule real-time sequencing and methylation-sensitive restriction enzymes, we successfully predicted putative motifs methylated by XvDMT1 and XvDMT2, which are previously uncharacterized 6mA and 5mC DNA MTases, respectively. This study provided new insights into the biological functions of DNA MTases in prokaryotic organisms.

## Introduction

*Xanthomonas euvesicatoria* (*Xe*), also known as *X*. *campestris* pv. *vesicatoria*, causes bacterial leaf spot disease, which is one of the most serious plant diseases worldwide that leads to serious damages and economic losses in plantations of tomato and pepper (Potnis et al., 2015). In particular, *Xe* is known to mostly invade leaves through stomata and wounded sites, subsequently colonizing intercellular spaces. Symptoms of the disease include the emergence of raised brown spots on leaves, stems, and fruits (Black et al., 2001). The *Xe*–tomato/pepper interaction has been widely used as a model system to study virulence mechanisms at the molecular level (Büttner et al., 2003). Using this pathosystem, most studies have focused on elucidating the functions of diverse type III effectors (Boch et al., 2009; Scheibner et al., 2017). However, other biological mechanisms in *Xe* have not been extensively characterized.

DNA methyltransferases (MTases) are known to catalyze the transfer of a methyl group from S-adenosyl-l-methionine (SAM) to nucleotides. Genome methylation by DNA MTases has been extensively studied in eukaryotic organisms and is known to be involved in their development and gene expression, and post-translational modifications (Du, 2016; Lyko, 2018). In *Arabidopsis thaliana*, DNA MTases and DNA demethylases have been antagonistically associated with immune responses to pathogen infection (Lopez Sanchez et al., 2016). Eukaryotic DNA MTases mostly add methyl groups on the fifth carbon of cytosine (5mC) (Smith and Meissner, 2013). In addition to 5mC, N^4^-methylcytosine (4mC) and N^6^-methyladenine (6mA) are also predominantly found in bacterial genomes (Sanchez-Romero et al., 2015). In contrast to eukaryotic DNA MTases, which have low genome recognition-site specificity, prokaryotic DNA MTases mostly recognize specific nucleotide sequences (Furuta et al., 2014). For example, DNA adenine MTase (DAM), the best-characterized DNA MTase, recognizes the GATC sequence, producing GmATC in *Escherichia coli* (Brooks et al., 1983). Accordingly, using single-molecule real-time (SMRT) sequencing and methylome analysis at the genome level, specific methylation motifs for DNA MTases have been successfully predicted in *Campylobacter coli, Helicobacter pylori, Ralstonia solanacearum, X. oryzae* pv. *oryzicola, X*. *axonopodis* pv. *glycines* (*Xag*), and *Xe* (Lee et al., 2015; Zautner et al., 2015; Seong et al., 2016; Erill et al., 2017; Xiao et al., 2018).

Bacterial DNA MTases are part of the restriction-modification (RM) system that protects bacteria against invasion by foreign DNA (Naito et al., 1995). Furthermore, in animal-associated bacteria, it has been known that functions of DNA MTases are associated with diverse biological mechanisms, including virulence, cell cycle, transcription regulation, and cell wall/envelope biogenesis (Heithoff et al., 1999; Reisenauer et al., 1999; Casadesus and Low, 2006; Marinus and Casadesus, 2009). For instance, M2.HpyAII, which recognizes the TCTTC site in *H. pylori*, is associated with transcription regulation and bacterial pathogenesis (Kumar et al., 2018). Likewise, the adenine MTase-overexpressing *Photorhabdus luminescens* exhibits decreased motility and differential gene expression compared with the wild-type strain (Payelleville et al., 2017). Park et al. (2019) recently characterized the roles of the EadM putative DNA MTase in *Xag* using phenotypic and proteomic analyses. Overexpression of EadM was shown to lead to reduced virulence, cell viability, and stress tolerance. Except for those of EadM, the functions of DNA MTases in plant-pathogenic bacteria are poorly understood.

In this study, we characterized the functions of two putative DNA MTases, XvDMT1 (*Xanthomonas euvesicatoria D*NA *MT*ase *1*; accession No., AOY65731) and XvDMT2 (accession No., AOY65938) in the *Xe* strain 85-10, for which both genome and methylome information have been reported (Seong et al., 2016). To elucidate the roles of these two DNA MTases in *Xe*, we generated XvDMT1- and XvDMT2-overexpressing strains, *Xe*(XvDMT1) and *Xe*(XvDMT2), respectively. To further investigate biological processes involving XvDMT1 and XvDMT2, we used label-free shotgun comparative proteomics and clusters of orthologous groups (COGs) classification. Based on the proteomic analysis, the roles of these two MTases were investigated using diverse phenotypic analyses, including virulence tests. Finally, putative motifs recognized by XvDMT1 and XvDMT2 were identified using SMRT sequencing and a DNA fragment assay, respectively.

## Materials and Methods

### Bacterial Strains and Growth Conditions

All bacterial strains used in this study are listed in Supplementary Table 1. *Xe* strains were grown in TS-broth (Tryptic Soy Broth Soybean-Casein Digest), XVM2 (20 mM NaCl, 10 mM (NH_4_)_2_SO_4_, 5 mM MgSO_4_, 1 mM CaCl_2_, 0.16 mM KH_2_PO_4_, 0.32 mM K_2_HPO_4_, 0.01 mM FeSO_4_, 10 mM fructose, 10 mM sucrose, and 0.03 % casamino acids (pH 6.7)) (Wengelnik et al., 1996), or M9 (240 mM Na_2_HPO_4_·7H_2_O, 110 mM KH_2_PO_4_, 43 mM NaCl, 93 mM NH_4_Cl, 2 mM MgSO_4_, and 0.1 mM CaCl_2_) media at 28°C with shaking (220 rpm). The DH5α *E. coli* strain, used for cloning, was grown in LB (1 % tryptone, 0.5 % yeast extract, and 1 % NaCl) at 37°C. Rifampicin (50 μg/mL), gentamicin (10 μg/mL), and ampicillin (100 μg/mL) were the antibiotics used for selection in this study.

### Generation of XvDMT-Overexpressing Strains

All plasmids used in this study are listed in Supplementary Table 1. To generate the constructs for *Xe*(XvDMT1) and *Xe*(XvDMT2), the open reading frame from genomic DNA was amplified using specific primers as follows: XvDMT1 F- 5′-ctcgagatgaacgccgtagaaatcga-3′, R- 5′-aagcttcagtggtggtggtggtggtgtgcgttggctcccaccttgc-3′, XvDMT2 F- 5′-ctcgagatgaacccgcaacctcgcac-3′, and R- 5′-aagcttcagtggtggtggtggtggtgatccgaagcggttgagcccg-3′. The amplified fragments were cloned into the pGem T-easy vector (Promega, Madison, WI, USA), and the sequence was confirmed using Sanger sequencing. Confirmed fragments were digested using *Xho*I and *Hin*dIII restriction enzymes, and the digested fragments were cloned into pBBR1-MCS5, which is the broad host-range vector and contains a *lac* promoter (Kovach et al., 1995), generating pMCS5-XvDMT1 and pMCS5-XvDMT2, respectively. The constructs were introduced into wild-type *Xe* using a MicroPulser^TM^ electroporator (Bio-Rad, Hercules, CA, USA), and transformed colonies were selected on TSA plates containing gentamicin. Selected XvDMT-overexpressing strains were confirmed using PCR analysis. In addition, an empty vector was introduced into *Xe*, generating *Xe*(EV), which was used as a negative control.

### Virulence Assay

*Solanum lycopersicum* cv. VF36 plants were grown in controlled chambers for 4 weeks at 26°C ± 1°C with a 16-h day/8-h night photoperiod. To prepare inocula, *Xe* strains were grown in TSA for 48 h, adjusted to an OD of 0.3 at 600 nm (OD_600_) in 10 mM MgCl_2_, corresponding to 10^8^ colony-forming units (CFU)/mL, and then diluted (10^−3^). The inoculum (about 10^8^ CFU/mL) was infiltrated into the third leaf using needleless syringes (Schaad et al., 1996). To measure bacterial growth, infiltrated leaves were punched with cork-borers (0.4 cm in diameter), and two leaf disk were ground in 200 μL of sterilized water using disposable micro pestles and 1.5 mL tubes. Extracted bacterial cells were serially diluted and dotted onto TSA-containing appropriate antibiotics rifampicin (50 μg/mL) and gentamicin (10 μg/mL). Three biological replicates were used for the assay.

### Label-Free Shotgun Proteomics

All procedures, including protein extraction and peptide preparation and conditions for liquid chromatography-tandem mass spectrometry (LC-MS/MS), have been previously described (Park et al., 2017). Briefly, three biological replicates of *Xe* strains were grown in XVM2 medium, which is a *hrp* inducing medium (Wengelnik et al., 1996), to an OD of 0.6 at 600 nm and harvested using centrifugation. After protein extraction and peptide generation, samples were analyzed using a split-free nano LC system (EASY-nLC II; Thermo Fisher Scientific, Waltham, MA, USA) connected to an LTQ Velos^TM^ instrument (Thermo Fisher Scientific, Rockford, IL, USA). Peptide identification and quantitation, comparison of protein abundance using statistical analysis, and clusters of orthologous groups (COGs) categorization were carried out as previously reported (Park et al., 2017). Briefly, the mass spectra obtained using LC-MS/MS were searched against the *Xe* strain 85-10 database from the National Center for Biotechnology Information, and peptides were quantitated using Thermo Proteome Discoverer 1.3 (ver. 1.3.0.399) combined with the SEQUEST program (Thermo Scientific). Data are available via ProteomeXchange with identifier PXD019554^1^. After identification and quantitation of peptides, protein abundance in *Xe*(XvDMT1) and *Xe*(XvDMT2) was compared with that in *Xe*(EV). Finally, differentially abundant proteins were classified using COGs analysis (Tatusov et al., 2000).

### Growth Analysis

To verify the effects of DNA MTases on bacterial growth, we monitored the growth patterns of the *Xe* strains in TSB, XVM2, and M9 media. Bacterial cultures were suspended in medium to an OD_600_ of 0.3 and diluted (10^−3^) (10^5^ CFU/mL). The OD_600_ was monitored for 8 d at 24-h intervals. To evaluate growth on different sugar sources, the *Xe* strains were grown in M9 medium containing 0.04 % glucose, sucrose, or fructose as the sole sugar source. Three biological replicates were used for the assay.

### Biofilm Analysis

To assess biofilm formation, *Xe* strains were cultured on TSA for 2 d. Cultured cells were washed with distilled water and resuspended to an OD_600_ of 0.3 (about 10^8^ CFU/mL). Then, bacterial suspensions were diluted 10^−1^ for 10^5^ CFU/mL and 10^−3^ for 10^7^ CFU/mL in TSB. For each strain, 190 μL of suspension was loaded in each well of a 96-well microplate (Costar, Kennebunk, ME, USA). After 5 d of incubation at 28°C without agitation, culture supernatants were removed using pipettes, and plates were washed with distilled water. The remaining cells were stained with 0.1 % crystal violet for 30 min. Stained cells were resuspended in 95 % EtOH, and the absorbance at 590 nm was measured using a microplate reader (SpectraMax plus 384; Molecular Devices, San Jose, CA, USA).

### EPS Analysis

To determine whether DNA MTases are involved in the production of EPS, we used a previously reported protocol (Underwood et al., 1995), with some modifications. *Xe* cells were harvested, diluted in TSB to an OD_600_ of 0.1, and incubated at 28°C for 5 d with shaking (220 rpm). Supernatants were collected using centrifugation at 15,300 × *g* for 15 min, and 400 μL of supernatant was mixed with 1.2 mL of EtOH. The mixture was placed at −20°C for 24 h. Then, the mixture was centrifuged at 16,500 × *g* at 4°C for 10 min, and the pellet was dried on a clean bench. Dried samples were mixed with 5 mL of sulfuric acid and 1 mL of aquaphenol (5 %), and the OD_488_ was measured using a spectrophotometer. Three biological replicates were used for the assay. For *E. coli* strains, a previously reported method was also used (Guo et al., 2010), with the sole exception that LB was used as the growth medium.

### Tolerance Assay

To estimate the roles of DNA MTases in stress tolerance, we used EtOH, d-sorbitol, and polymyxin B as stress factors (Li and Wang, 2011). *Xe* cells were harvested, washed, and diluted in TSB to 10^5^ CFU/mL. The *Xe* strains were incubated in TSB with 2 % EtOH for 4 d in a shaking incubator at 28°C. As for d-sorbitol and polymyxin B, bacterial suspensions were grown in TSB to an OD_600_ of 0.1. The *Xe* strains were exposed to 40 % d-sorbitol for 20 min and 75 ng/mL of polymyxin B for 2 h in TSB. After treatments, cultures were serially diluted with sterilized water, and bacterial numbers were determined using a colony counting method. Cell survivability was calculated as the ratio of the bacterial number after exposure to TSB without stress factors and the number after exposure to a given stress factor. For *E. coli* strains, the conditions used were identical to those for *Xe* strains except that the LB medium was used for incubation.

### LPS Analysis

Bacterial cells were incubated at 28°C for *Xe* and 37°C for *E. coli*. When bacterial cells reached an OD_600_ value of 0.8, 5 mL of culture was collected. Subsequently, LPSs were extracted using the LPS extraction kit (iNtRON biotechnology, Seongnam, Republic of Korea), separated by tricine-sodium dodecyl sulfate polyacrylamide gel electrophoresis, and then visualized using a Pierce silver staining kit (Thermo Fisher Scientific).

### Methylation Analysis

For methylome analysis, we used the ER3413 *E*. *coli* strain, which is a DNA MTase-deficient strain (Anton et al., 2015). The pMCS5, pMCS5-XvDMT1, and pMCS5-XvDMT2 plasmids were introduced into ER3413, generating ER3413(EV), ER3413(XvDMT1), and ER3413(XvDMT2), respectively, and transformants were selected on LB plates containing gentamicin. Colonies were confirmed using PCR analysis and MCS5 primers, and XvDMT-specific primers. In addition, we also used *Xe*(EV), *Xe*(XvDMT1) and *Xe*(XvDMT2) strains. Genomic DNA was extracted from cells grown to an OD_600_ of 0.6 using a genomic DNA extraction kit (Qiagen, Hilden, Germany). SMRT sequencing and bioinformatics analysis were conducted as previously described (Seong et al., 2016). Briefly, 5 μg of genomic DNA from each strain was used, and SMRTbell template libraries were generated using the SMRTbell™ Template Prep Kit 1.0 (Pacific Biosciences, Menlo Park, CA, USA) following the manufacturer's instructions. Libraries were sequenced using 1 SMRT cell (Pacific Biosciences) and C4 chemistry (DNA sequencing Reagent 4.0) (Varela-Alvarez et al., 2006). Raw data generated by PacBio sequencing were deposited to the NCBI Sequence Read Archive under BioProject accession ID PRJNA670940. The sequencing reads of the three strains were assembled using the Hierarchical Genome Assembly Process (version 2.3) (Chin et al., 2013). The base modification was identified using SMRT Analysis 1.1 and RS_Modification (Pacific Biosciences). The 6mA and 4mC were identified using ipdSummary (Pacific Biosciences), where the further analysis was performed using over 10 of base coverages and over 30 of quality values. Motif identification was accomplished using MotifMaker (https://github.com/PacificBiosciences/MotifMaker) and in-house Python scripts with REBASE version 903 (ftp://ftp.neb.com/pub/rebase/nar.903.txt). Wildtype *Xe* was reanalyzed using previously reported raw data (Seong et al., 2016).

### DNA Fragment Assay

To assess the methylation ability of the putative DNA MTases, *Xe*(EV), *Xe*(XvDMT2), and *Xe*(XvDMT2) were grown in XVM2 medium to an OD_600_ of 0.6, and bacterial cells were harvested using centrifugation. Genomic DNA was extracted using a genomic DNA extraction kit (Qiagen). The DNA quantities and quality were checked by NanoDrop One (Thermo Scientific). The genomic DNA (1000 ng) was digested for 15 min using 2.5 units of *Msp*JI (NEB, Ipswich, MA, USA), McrBC (NEB), *Eco*RII (Thermo Scientific), and *Bsp*119I (Thermo Scientific) at the optimal incubation temperature for each enzyme. The digested genomic DNA was electrophoresed in a 1 % agarose gel at 100 V for 90 min.

### Statistical Analysis

All experiments were repeated at least three times using three replicates. Statistical analyses were carried out using Student's *t*-test and one-way analysis of variance combined with Tukey's multiple comparison using the SPSS 12.0K software (SPSS, Inc., Chicago, IL, USA). *P* < 0.05 was considered significant.

## Results

### Overexpression of XvDMT2, but Not XvDMT1, Reduced *Xe* Virulence in Tomato

**Figure 1:**
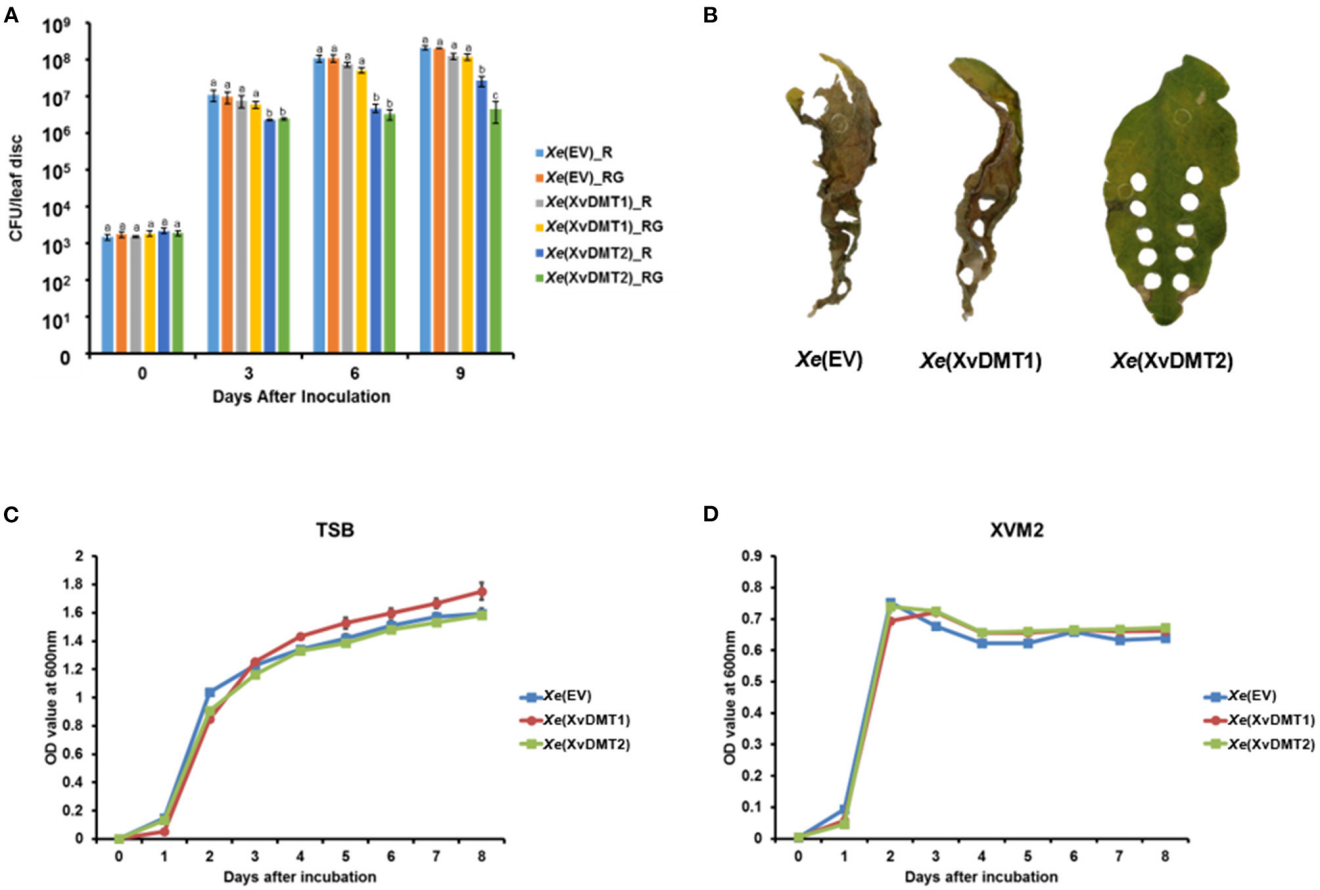
Virulence assay of the *Xe* strains in plants. Suspensions of *Xe*(EV), *Xe*(XvDMT1), and *Xe*(XvDMT2) strains (10^5^ cells/mL) were infiltrated into tomato leaves using a needleless syringe. **(A)** Growth of *Xe*(EV), *Xe*(XvDMT1), and *Xe*(XvDMT2) was evaluated using the colony counting method on two different plates (R, TSA with rifampicin and RG, TSA with rifampicin and gentamycin) at 0, 3, 6, and 9 DAI. Different letters represent significant differences (*P* ≤ 0.05; one-way analysis of variance followed by Tukey's multiple). Error bars indicate standard deviations. **(B)** Photographs of tomato leaves infected by the three strains, taken at 12 DAI. Bacterial growth of *Xe*(EV), *Xe*(XvDMT1), and *Xe*(XvDMT2) in **(C)** TSB and **(D)** XVM2 medium supplemented with rifampicin (50 μg/mL) and gentamicin (10 μg/mL) was monitored for 8 d at 24-h intervals. Experiments were repeated at least three times, with three biological replicates. All experiments were repeated at least three times with three replicates.

Most bacterial DNA MTases are classified into SAM-dependent family, generating N6-methyladenine (6mA), N4-methylcytosine (4mC), and C5-methylcytosine (5mC) (Sanchez-Romero et al., 2015). The deduced amino acid (AA) sequences of XvDMT1 (926 aa) and XvDMT2 (538 aa) are known to display high similarity with those of putative SAM-dependent DNA MTases and putative cytosine DNA MTases, respectively, in diverse gram-negative bacteria (Supplementary Figures 1A,B), but are not very similar between themselves (Supplementary Figure 1C). Therefore, it could be postulated that XvDMT1 and XvDMT2 have distinct functions. Although we tried to establish *xvDMT*-null mutants to elucidate the roles of XvDMT1 and XvDMT2, the mutants were not generated with many attempts. Therefore, we generated XvDMT1- and XvDMT2-overexpressing strains, namely *Xe*(XvDMT1) and *Xe*(XvDMT2), respectively. The *Xe* strain possessing an empty vector, termed *Xe*(EV), was used as a control to eliminate vector effects. To test the potential roles of XvDMT1 and XvDMT2 in virulence, *Xe*(EV), *Xe*(XvDMT1), and *Xe*(XvDMT2) were infiltrated into tomato leaves. Bacterial growth in infected leaves was determined at 0, 3, 6, and 9 d after inoculation (DAI) using a colony counting method. To check the stability of the plasmids in the *Xe* strains, we analyzed the dotted bacterial population following incubation on tryptic soy agar (TSA) with rifampicin (R) and TSA with rifampicin and gentamycin (RG). As shown in Figure 1A, the numbers of viable cells of *Xe*(XvDMT1) and *Xe*(EV) were not significantly different. In contrast, the numbers of viable cells of *Xe*(XvDMT2) were observed to be considerably lower than those of *Xe*(EV) at 3, 6, and 9 DAI (Figure 1A). We did not observe any differences in the numbers of bacterial cells among the three strains between the two plating conditions at 0, 3, and 6 DAI. The numbers of *Xe*(XvDMT2) were much lower than those of *Xe*(EV) and *Xe*(XvDMT1) in both plates. At 9 DAI, the numbers of *Xe*(XvDMT2) grown in TSA_R were a little higher than those in TSA_RG, suggesting partial loss of the XvDMT2 plasmid by *Xe*(XvDMT2) at that time point. Leaves infected by *Xe*(EV) and *Xe*(XvDMT1) showed apparent disease symptoms (Figure 1B). In contrast, leaves infected by *Xe*(XvDMT2) remained green (partially yellow) and maintained their typical shapes, demonstrating that overexpression of XvDMT2 partially suppressed the virulence of *Xe*. Therefore, it could be postulated that the reduced virulence of *Xe*(XvDMT2) was due to the overexpression of XvDMT2. Next, we examined bacterial growth in rich (TSB) and virulence factor-inducing (XVM2) media. In TSB, the growth of the three strains was not significantly different (Figure 1C). Similarly, there was no considerable difference observed in the growth among the strains in XVM2 (Figure 1D). These results indicated that the reduced growth of *Xe*(XvDMT2) in tomato leaves was not caused by an effect on bacterial growth or reproduction.

### Comparative Proteomic Analysis for XvDMTs

Overexpression of XvDMT2 led to reduced virulence; however, the other biological processes associated with XvDMTs remained unclear. Therefore, we carried out a label-free shotgun comparative proteomic analysis followed by cluster of orthologous groups (COGs) classification. Accordingly, protein abundances in *Xe*(XvDMT1) and *Xe*(XvDMT2) were compared with those in *Xe*(EV).

The numbers of proteins detected in biological replicates for all three strains are shown in Supplementary Table 2. In particular, *Xe*(EV), *Xe*(XvDMT1), and *Xe*(XvDMT2), respectively, were demonstrated to have 993, 1109, and 1144 proteins in common in the three biological replicates. In all independent strains, *Xe*(EV) had fewer proteins in common than *Xe*(XvDMT1) and *Xe*(XvDMT2). However, the numbers of peptide spectral matches (PSMs) were not highly variable in the three biological replicates of each strain (Supplementary Table 2), indicating an efficient proteomic analysis. Because we simultaneously prepared and analyzed all biological samples under identical conditions, we used the commonly found proteins in the three biological replicates from each strain for comparative proteomic analysis to report unbiased results.

**Figure 2:**
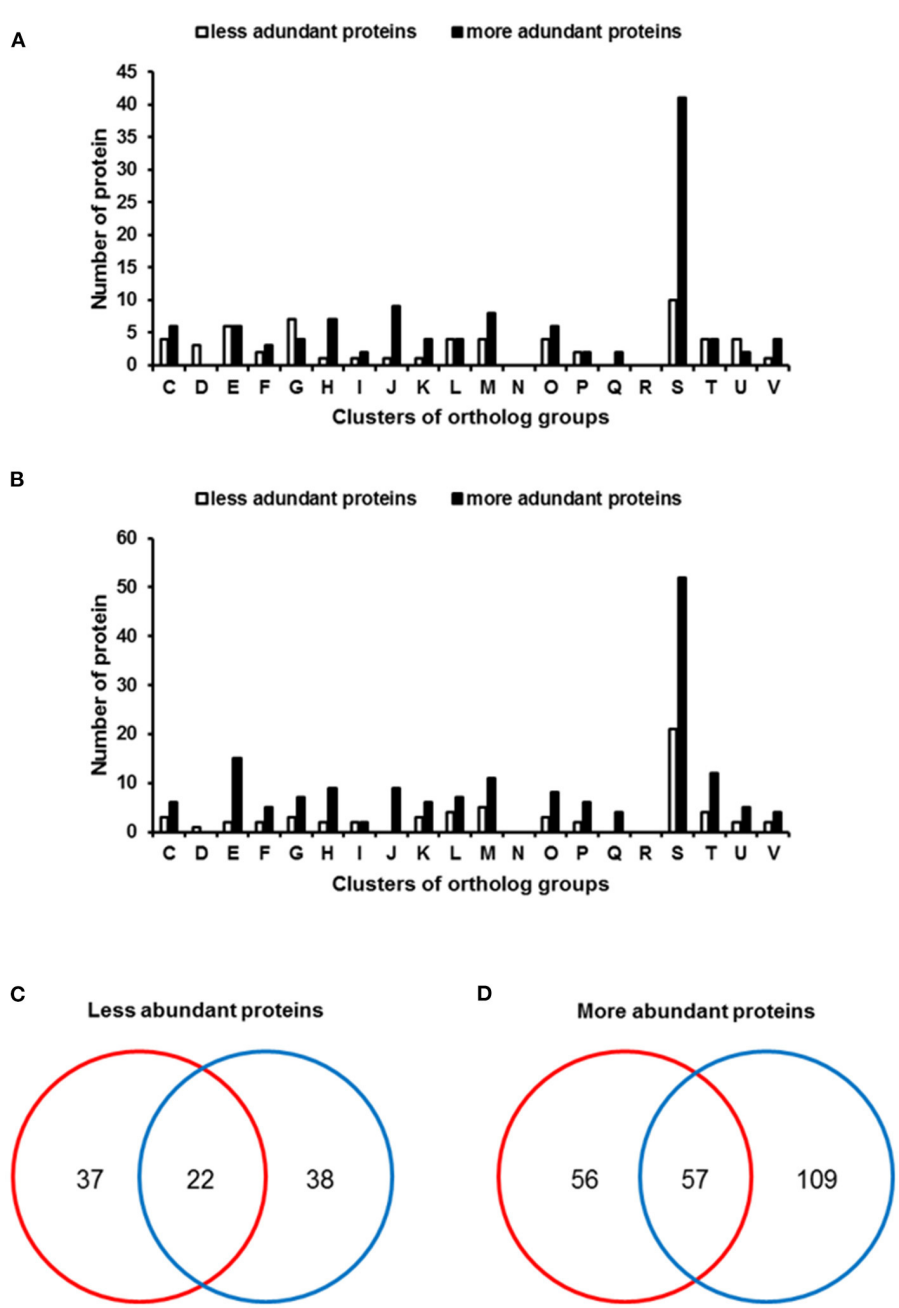
Clusters of orthologous group (COGs) analysis of differentially abundant proteins in *Xe* strains. Bars represent COGs that were differentially abundant (>2-fold) in **(A)** *Xe*(XvDMT1) and **(B)** *Xe*(XvDMT2). Venn diagrams reveal numbers of proteins that were **(C)** less or **(D)** more abundant in *Xe*(XvDMT1) (red) and *Xe*(XvDMT2) (blue). Functional groups: C, energy production and conversion; D, cell cycle control and mitosis; E, amino acid metabolism and transport; F, nucleotide metabolism and transport; G, carbohydrate metabolism and transport; H, coenzyme metabolism; I, lipid metabolism; J, translation; K, transcription; L, replication and repair; M, cell wall/membrane/envelop biogenesis; N, cell motility; O, post-translational modification, protein turnover, chaperone functions; P, inorganic ion transport and metabolism; Q, secondary structure; R, general functional prediction only; S, function unknown; T, signal transduction; U, intracellular trafficking and secretion; V, defense mechanisms.

First, we compared proteins detected in *Xe*(XvDMT1) and *Xe*(EV). In total, 60 and 114 proteins were found to be more abundant (>2-fold) in *Xe*(EV) and *Xe*(XvDMT1), respectively (Supplementary Tables 3, 4). As expected, XvDMT1 was more abundant (>47-fold) in *Xe*(XvDMT1) (Supplementary Table 4), confirming its overexpression in *Xe*(XvDMT1). Differentially abundant proteins were categorized by COGs analysis (Figure 2A). Except for those in groups D (cell cycle control and mitosis), G (carbohydrate metabolism and transport), and U (intracellular trafficking and secretion), protein numbers in *Xe*(EV) were lower than those in *Xe*(XvDMT1) for most categories. The abundance of proteins in groups E (energy production and conversion), G (carbohydrate metabolism and transport), and M (cell wall/membrane/envelop biogenesis) was strongly affected by XvDMT1.

Next, we compared proteins in *Xe*(XvDMT2) and *Xe*(EV); 61 and 167 proteins were identified to be more abundant (>2-fold) in *Xe*(EV) and *Xe*(XvDMT2), respectively, than in the other strain, and were subjected to COGs analysis (Supplementary Tables 5, 6). In addition, XvDMT2 was identified only in *Xe*(XvDMT2), with 226 PSMs under the given conditions (Supplementary Table 5), indicating that *Xe*(XvDMT2) indeed overexpressed XvDMT2. Except for group D (cell cycle control and mitosis), for most categories, protein numbers were higher in *Xe*(XvDMT2) than in *Xe*(EV) (Figure 2B). Notably, proteins categorized in group N (motility) were not identified in either of the comparisons, suggesting that XvDMT1 and XvDMT2 were not associated with movement in *Xe*. In addition, proteins belonging to group D were found only in *Xe*(EV) in both comparisons.

Next, we compared proteins identified in the proteomic analysis for *Xe*(XvDMT1) and *Xe*(XvDMT2) (Figures 2C,D). We found that 22 proteins were less abundant in both *Xe*(XvDMT1) and *Xe*(XvDMT2) compared with those in *Xe*(EV), with the abundance of 37 and 38 proteins being negatively affected by the overexpression of XvDMT1 and XvDMT2, respectively (Figure 2C and Supplementary Table 7). Among the proteins that were more abundant in *Xe*(XvDMT1) and *Xe*(XvDMT2) relative to *Xe*(EV), 57 were identified in both strains (Figure 2D and Supplementary Table 8). More specifically, 56 and 109 proteins were found in *Xe*(XvDMT1) and *Xe*(XvDMT2), respectively. These data suggested that XvDMT1 and XvDMT2 were largely involved in different biological mechanisms in *Xe*, although some processes might require both enzymes. Notably, the numbers of the more abundant proteins in XvDMT1 and XvDMT2 were higher than those of the less abundant proteins. It could be thus speculated that the numbers of shared proteins in the three biological replicates of *Xe*(XvDMT1) and *Xe*(XvDMT2) were higher than those in the replicates of *Xe*(EV). Therefore, we mainly focused on the proteins that were less abundant in *Xe*(XvDMT1) and *Xe*(XvDMT2) in subsequent phenotypic assays to characterize the functions of XvDMT1 and XvDMT2.

### XvDMT-Overexpressing Strains Showed Growth Retardation in Sucrose or Fructose as the Sole Carbon Source

**Figure 3:**
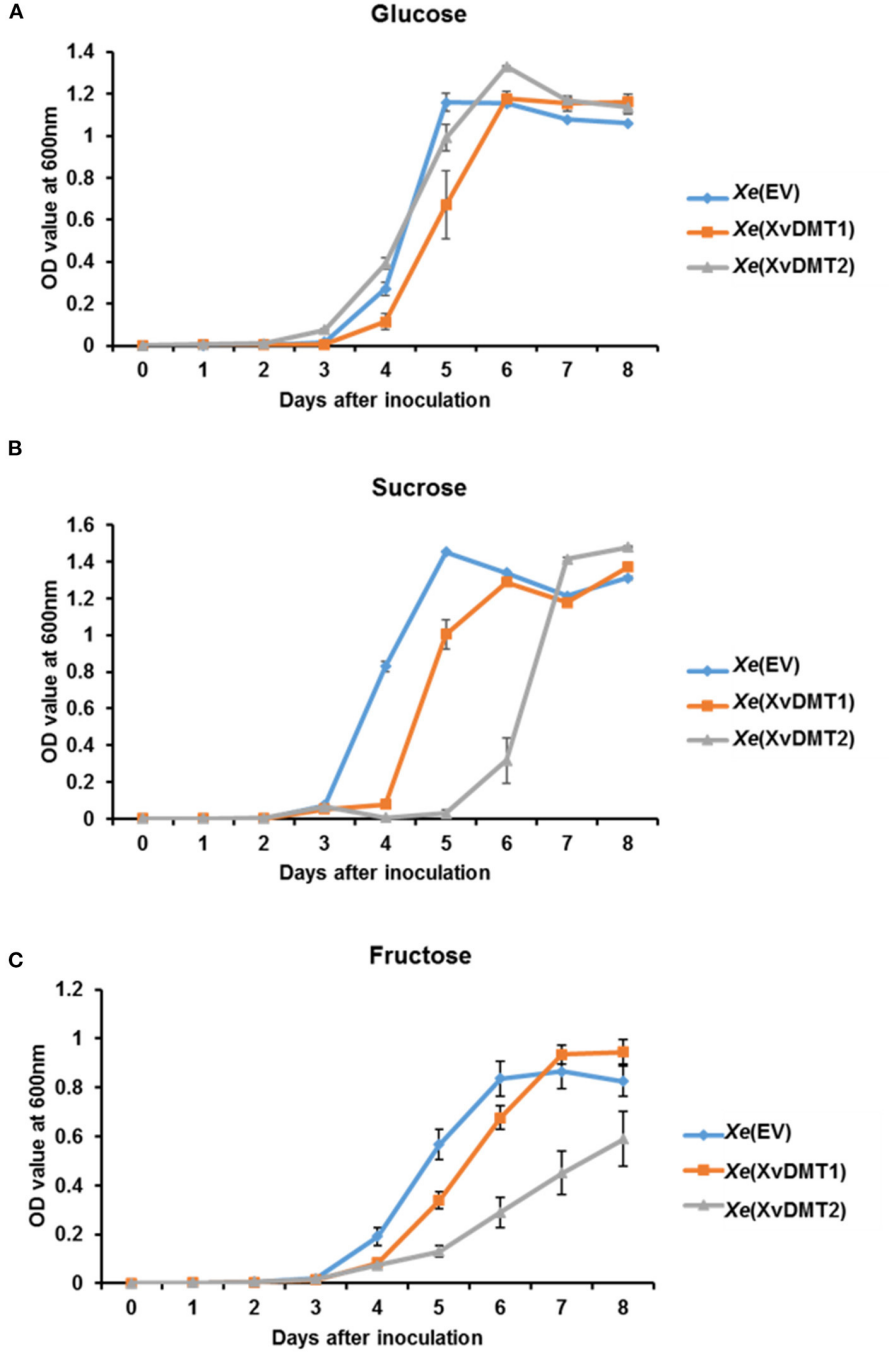
Bacterial growth in M9 medium in the presence of different sugars as a carbon source. *Xe*(EV) (blue), *Xe*(XvDMT1) (orange), and *Xe*(XvDMT2) (gray) strains were grown in M9 medium supplemented with 0.4 % **(A)** glucose, **(B)** sucrose, or **(C)** fructose as the sole sugar source. Rifampicin (50 μg/mL) and gentamicin (10 μg/mL) were also added. Bacterial growth was monitored for 8 d at 24-h intervals. Error bars represent standard deviations. All experiments were repeated at least three times with three replicates.

The comparative proteomic analysis revealed that the abundance of proteins related with the sugar transport systems was altered following the overexpression of XvDMT1 or XvDMT2. Particularly, the phosphocarrier protein HPr, which is involved in sugar uptake through a group translocation system (Deutscher et al., 2006), was shown to be less abundant in both *Xe*(XvDMT1) and *Xe*(XvDMT2) strains (Supplementary Table 7). Therefore, we investigated the growth ability of *Xe*(EV), *Xe*(XvDMT1), and *Xe*(XvDMT2) in M9 medium supplemented with 0.4 % glucose, sucrose, or fructose as the sole carbon source. Accordingly, in the presence of glucose, the growth of all three strains was generally similar, although the growth of *Xe*(XvDMT1) was slightly slow (Figure 3A). Growth increased dramatically after 3 d and steadily decreased after 6 d of incubation. In contrast, when sucrose was used as a carbon source, the growth of *Xe*(XvDMT1), and especially that of *Xe*(XvDMT2), was retarded (Figure 3B). The growth of *Xe*(XvDMT1) was elevated 1 d later than that of *Xe*(EV), whereas *Xe*(XvDMT2) started to grow 2 d later than *Xe*(EV). In the presence of fructose, *Xe*(EV) showed delayed growth compared with that on glucose or sucrose (Figure 3C). In addition, the growth of *Xe*(XvDMT2) in the presence of fructose was significantly slower than that of *Xe*(EV). Of note, the growth of all three strains in TSB and XVM2 did not considerably differ (Supplementary Figures 2B,C), indicating that XvDMT1 and XvDMT2 have functions in sugar metabolism or transport, but not in bacterial multiplication or reproduction.

### Overexpression of XvDMT2 Showed Reduced Biofilm Formation and Exopolysaccharide Production

**Figure 4:**
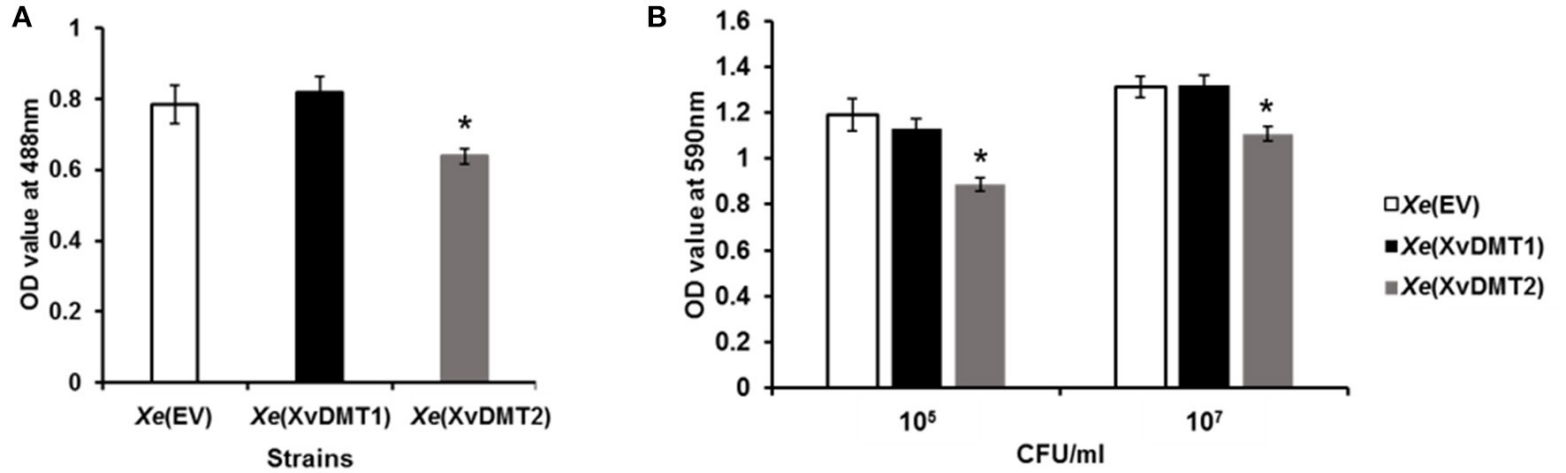
Production of EPSs and formation of biofilm in *Xe* strains. **(A)** EPS production and **(B)** biofilm formation in *Xe*(EV) (white), *Xe*(XvDMT1) (black), and *Xe*(XvDMT2) (gray) were measured using a phenol-sulfuric acid method and crystal violet staining, respectively. Biofilm formation was evaluated with two (about 10^5^ and 10^7^ CFU/mL) of initial populations. Error bars represent standard deviations. *P ≤ 0.05 (Student's *t*-test). All experiments were repeated at least three times with three replicates.

The abundance of proteins related to the biogenesis of the cell wall/membrane/envelope and metabolism/transport of carbohydrates were demonstrated to be mostly altered in the comparative proteomic analysis (Figures 2A,B), with *Xe*(XvDMT2) displaying reduced virulence (Figure 1A). It is well known that the production of exopolysaccharide (EPS), which are secreted at and associated with the cell surface, is affected by sugar sources and is related to bacterial virulence in *Xanthomonas* spp. (Looijesteijn et al., 1999; Bae et al., 2018b). Therefore, we evaluated the production of EPSs in *Xe*(XvDMT1) and *Xe*(XvDMT2) by quantitating carbohydrates using a previously reported method (Park et al., 2019). The production of EPSs by *Xe*(XvDMT2), but not *Xe*(XvDMT1), was shown to be significantly lower than that observed in *Xe*(EV) (Figure 4A), indicating that XvDMT2, but not XvDMT1, was involved in the production of EPSs. Biofilm formation, whereby bacteria adhere to a surface and protect themselves, has been reported to be closely related to EPSs (Sutherland, 2001). Thus, we investigated the ability of biofilm formation in the three strains using a 96-well plate assay. Similar to the production of EPSs, the ability of *Xe*(XvDMT2) for biofilm formation was significantly lower than that of *Xe*(EV) (Figure 4B). In contrast, biofilm formation by *Xe*(XvDMT1) was comparable to that observed in *Xe*(EV). This result suggested that XvDMT2 in *Xe* was involved in biofilm formation or adhesion in the given condition.

### XvDMTs Were Related With Tolerance to Ethanol, Sorbitol, and Polymyxin B

**Figure 5:**
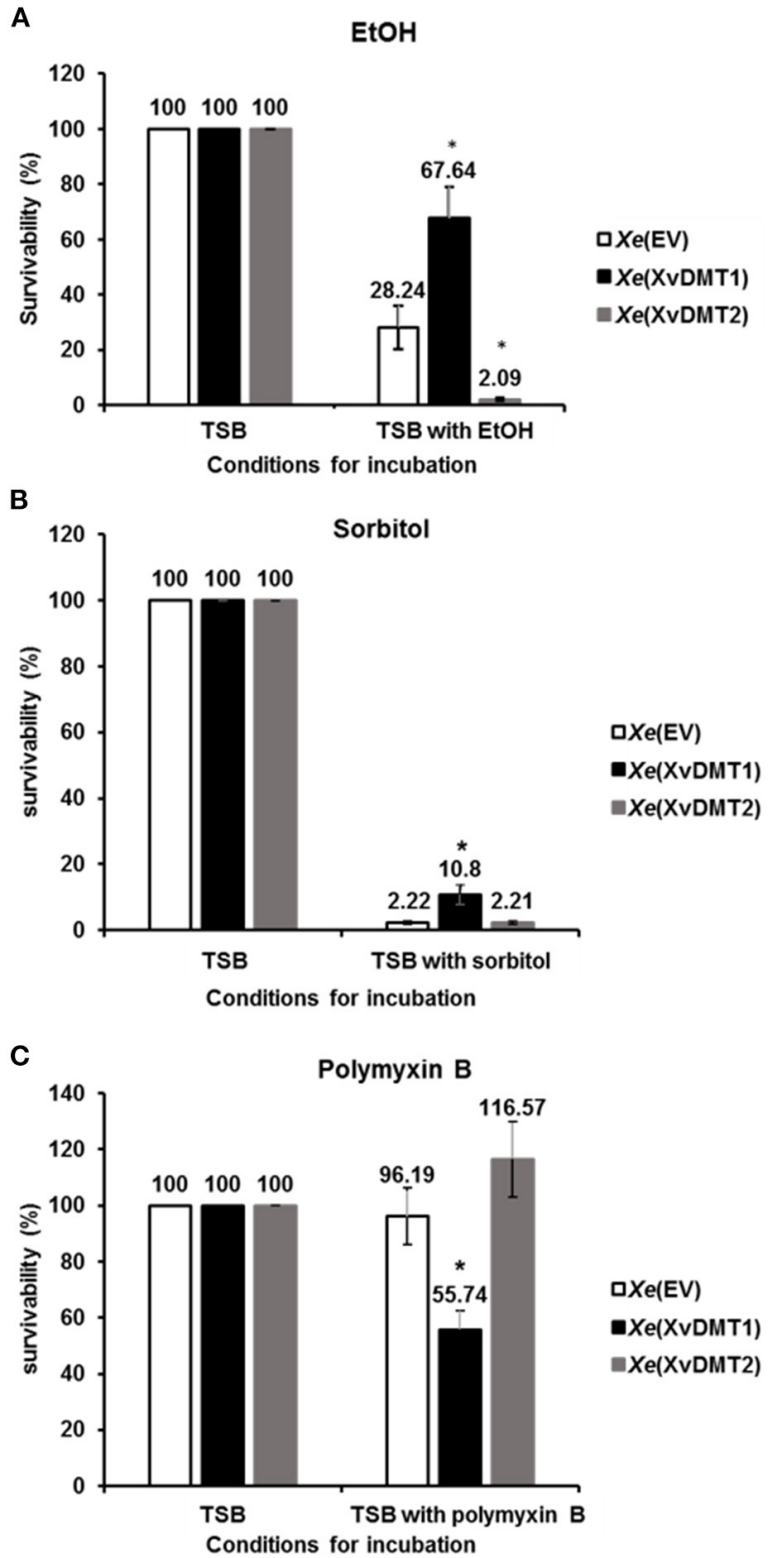
Tolerance to EtOH, sorbitol, and polymyxin B. *Xe*(EV) (white), *Xe*(XvDMT1) (black), and *Xe*(XvDMT2) (gray) were exposed to **(A)** 2 % EtOH for 4 d **(B)** 40 % sorbitol for 20 min, or **(C)** 75 ng/mL of polymyxin B for 2 h. Survival was assessed using colony counting. Survivability was calculated as the ratio of viable cell numbers in TSB and viable cell numbers in TSB with stress factors. Error bars represent standard deviations. *P ≤ 0.05 (Student's *t*-tests). All experiments were repeated at least three times with three replicates.

Because the abundance of cell wall/membrane/envelop-related proteins was affected by the overexpression of XvDMT2 or XvDMT1 (Supplementary Table 7), we evaluated the tolerance of *Xe*(XvDMT1) and *Xe*(XvDMT2) to various stresses in comparison with that of *Xe*(EV). As shown in Figure 5A, in the presence of 2% ethanol (EtOH), which has adverse effects on the integrity of the bacterial membrane (Ingram, 1990), the survivability of *Xe*(XvDMT1) and *Xe*(XvDMT2) was significantly higher (67.64 %) and lower (2.09 %), respectively, compared with that of *Xe*(EV). It suggests that both proteins had opposite functions in the tolerance of *Xe* to EtOH. When *Xe* strains were exposed to 40% sorbitol as an osmotic agent, the survivability of *Xe*(XvDMT1) was significantly increased compared with, whereas that of *Xe*(XvDMT2) was comparable to that of *Xe*(EV) (Figure 5B). These results indicate that *Xe*(XvDMT1) is more tolerant to osmotic stress than *Xe*(EV). In the presence of 0.075 μg/mL polymyxin B, which binds to bacterial membranes and exerts bactericidal activity (Landman et al., 2008), survivability did not significantly differ between *Xe*(EV) and *Xe*(XvDMT2) (Figure 5C). Notably, *Xe*(XvDMT1) was substantially more sensitive to polymyxin B than *Xe*(EV).

### XvDMT1 and XvDMT2 Are Site-Specific DNA MTases

**Figure 6:**
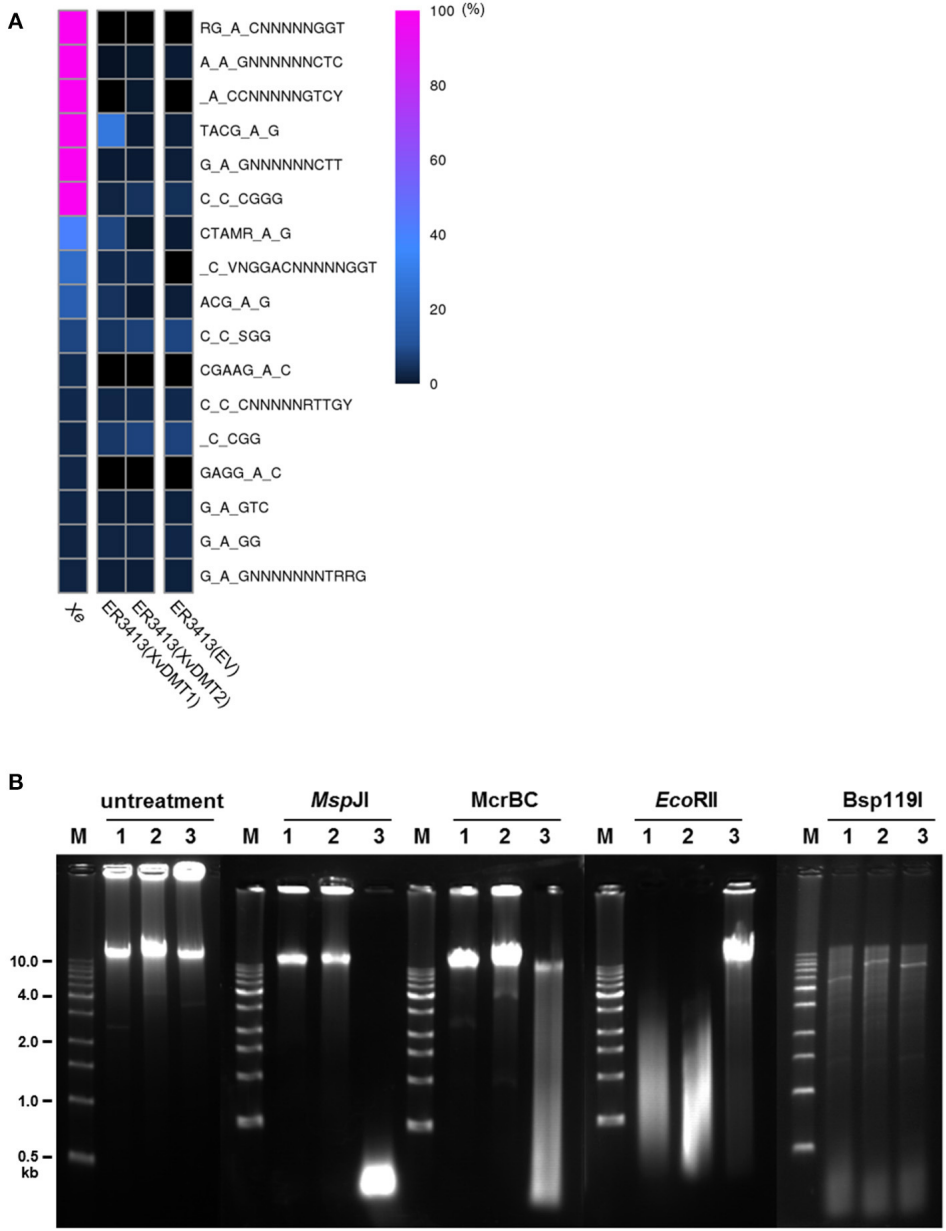
Prediction of putative methylation motifs. **(A)** Heatmap of methylation at putative methylation motifs based on methylome analyses of *Xe*, ER3413(EV), ER3413(XvDMT1), and ER3413(XvDMT2) using SMRT sequencing. *Xe* is the wild-type strain. ER3413(EV), ER3413(XvDMT1), and ER3413(XvDMT2) are ER3413 *E*. *coli* strains carrying the pBBR1-MCS5, pMCS5-XvDMT1, and pMCS5-XvDMT2 plasmids, respectively. Purple and black indicate 100 and 0% methylation in the motifs, respectively. **(B)** Patterns of genomic DNA fragments digested by three restriction enzymes in *Xe* strains. Extracted genomic DNA from *Xe*(EV) (1), *Xe*(XvDMT1) (2), and *Xe*(XvDMT2) (3) was treated with *Msp*JI, McrBC, *Eco*RII, or Bsp119I. M: 1-kb DNA ladder.

Using SMRT genome sequencing, we previously reported putative motifs methylated by putative 6mA and 4mC DNA MTases in *Xe* str. 85-10 (Seong et al., 2016). Here, we aimed to predict putative methylation motifs for XvDMT1 and XvDMT2 by SMRT sequencing using *E. coli* str. ER3413, which is a DNA MTase-deficient strain (Lee et al., 2015). The pMCS5-XvDMT1 and pMCS5-XvDMT2 plasmids, which were used for creating the XvDMT-overexpressing *Xe* strains, were introduced into the ER3413 strain of *E. coli*, generating ER3413(XvDMT1) and ER3413(XvDMT2), respectively. In addition, ER3413(EV) was generated as a control for comparison. We analyzed putative methylated motifs in ER3413(XvDMT1), ER3413(XvDMT2), and ER4313(EV) using SMRT sequencing. For comparison, we also reanalyzed the wild-type strain's methylome using the raw data obtained in a previous report (Seong et al., 2016). In the *Xe* genome, six methylation motifs (RGmACNNNNNGGT, AmAGNNNNNNCTC, mACCNNNNNGTCY, TACGmAG, GmAGNNNNNNCTT, and CmCCGGG) were nearly fully methylated (>99 %) (Figure 6A and Supplementary Table 9). We first analyzed the methylomes of *Xe*(EV), *Xe*(XvDMT1), and *Xe*(XvDMT2) and found that the patterns of the six methylation motifs were not different among the three strains (Supplementary Figure 2), suggesting that overexpression of XvDMT1 or XvDMT2 did not change the status of methylation in *Xe*. Next, we analyzed the methylation patterns in ER3413(XvDMT1), ER3413(XvDMT2), and ER4313(EV). The six putative motifs identified in *Xe* were hardly methylated in ER3413(XvDMT2) and ER3413(EV) (Figure 6A and Supplementary Table 9). Notably, we detected methylation on the second adenine in TACGAG (27.2 %) in ER3413(XvDMT1) (Figure 6A and Supplementary Table 9). The IPD ratio and distribution of methylated fractions for TACGmAG in ER3413(XvDMT1) were different from those in ER3413(XvDMT2) and ER3413(EV) (Supplementary Figure 3). These data indicated that XvDMT1 is a site-specific 6mA DNA MTase. However, we did not identify any putative motifs targeted by XvDMT2 using SMRT sequencing.

XvDMT2 shows high similarity to putative cytosine DNA MTases (Supplementary Figure 1C), and it is challenging to detect 5mC using SMRT sequencing. Therefore, methylation-sensitive restriction enzymes were used to predict putative motifs for XvDMT2. To this end, genomic DNAs extracted from *Xe*(EV), *Xe*(XvDMT1), and *Xe*(XvDMT2) were digested using four site-specific, methylation-sensitive restriction enzymes. In addition, undigested genomic DNA from the three strains was used as a negative control. Accordingly, the fragmented patterns produced by *Msp*JI, McrBC, and *Eco*RII, which are 5mC-sensitive restriction enzymes in *Xe*(XvDMT2), were different from those in *Xe*(EV) and *Xe*(XvDMT1) (Figure 6B). In particular, *Msp*JI is known to recognize CNN(A/G) sites and cuts only methylated CNN(A/G), 5′-mCNNR-3′ (Cohen-Karni et al., 2011). We noted that *Msp*JI digested the genomic DNA from *Xe*(XvDMT2) but not those from *Xe*(EV) and *Xe*(XvDMT1), indicating that XvDMT2 is a 5mC DNA MTase. The restriction site recognized by McrBC is 5′-(A/G)mC(Nn)(A/G)mC-3′, and unmethylated sites are not cleaved (Sukackaite et al., 2012). McrBC was shown only to be able to cut the genomic DNA of *Xe*(XvDMT2). *Eco*RII is known to recognize and cleave CC(A/T)GG sequences, but digestion by *Eco*RII is blocked by methylation on the second C5 position (Dcm methylation) (Mucke et al., 2002). In our test, *Eco*RII did not digest the genomic DNA of *Xe*(XvDMT2), but it did digest the genomic DNA of *Xe*(EV) and *Xe*(XvDMT1) (Figure 6B). However, the patterns of fragmented genomic DNA cleaved by *Bsp*119I, which recognizes 5′-TTCGAA-3′ and is 5mC-sensitive, were not significantly different among the three strains (Figure 6B). These results suggested that XvDMT2 is a site-specific 5mC DNA MTase.

### Phenotypes of ER3413 Strains Were Not Changed by XvDMT1 or XvDMT2

To elucidate any pleiotropic effects of XvDMT1 or XvDMT2 in *E. coli*, we investigated the tolerance to EtOH, sorbitol, and polymyxin B and the production of EPSs using ER3413(EV), ER3413(XvDMT1), and ER3413(XvDMT2) (Supplementary Figure 4). Instead of TSB, Luria-Bertani (LB) was used for *E. coli*, whereas the other experimental conditions for *E. coli* strains were similar to those used for *Xe* strains. We did not observe any statistical difference among the three strains in any phenotypic experiments (Supplementary Figures 4A–D), indicating that XvDMT1 and XvDMT2 were specific to *Xe*. Next, we examined the profiles of lipopolysaccharides (LPSs) from ER3413(EV), ER3413(XvDMT1), and ER3413(XvDMT2), but noted that all three strains showed similar profiles (Supplementary Figure 4E). Finally, we also compared the LPS profiles of *Xe*(EV), *Xe*(XvDMT1), and *Xe*(XvDMT2) (Supplementary Figure 5F); the profile of *Xe*(EV) was not different from those of *Xe*(XvDMT1), and *Xe*(XvDMT2), suggesting that XvDMT1 or XvDMT2 were not involved in the formation of LPSs.

## Discussion

Bacterial DNA MTases have garnered increasing research interest. The functions of DNA MTases have been well characterized in animal pathogenic bacteria. For example, the CcrM DNA MTase has been shown to be indispensable for cell viability in *Brucella abortus* (Robertson et al., 2000). In *Klebsiella pneumoniae*, which causes liver abscess, metastatic endophthalmitis, and metastatic meningitis, adenine methylation by *dam* MTase has been associated with the regulation of its pathogenicity (Fang et al., 2017). However, the functions of DNA MTases in plant-pathogenic bacteria have not been well documented. Recently, it was reported that the functions of EadM were related to cell wall/envelopes, tolerance to various stresses, production of siderophore, and virulence in *Xag* (Park et al., 2019). Similarly, in this study, overexpression of XvDMT2, but not XvDMT1, was shown to reduce the virulence of *Xe*. It is generally recognized that DNA MTases play critical roles in bacterial growth and virulence by affecting replication. However, XvDMT1 and XvDMT2 were shown not to be involved in bacterial reproduction and replication.

Comparative proteomic analysis revealed that overexpression of XvDMT1 and XvDMT2 altered the abundance of proteins involved in the metabolism and transport of carbohydrates. Specifically, we found that the phosphocarrier protein HPr was negatively regulated by XvDMT1 and XvDMT2 (Supplementary Table 7). The HPr protein is a component of the phosphoenolpyruvate-dependent sugar phosphotransferase system (PTS) (Deutscher et al., 1995). The PTS is indispensable for transporting diverse carbohydrates, including glucose, fructose, and sucrose (Siebold et al., 2001). In *Xe*(XvDMT1) and *Xe*(XvDMT2), growth in the presence of sugar as the sole carbon source, specifically sucrose and fructose, was retarded (Figure 3). Therefore, it could be postulated that XvDMT1 and XvDMT2 might have functions in regulating the transport of carbohydrates mediated by the PTS. In particular, we observed that growth retardation in *Xe*(XvDMT2) was significantly more severe than that in *Xe*(EV) and *Xe*(XvDMT1), which also displayed reduced virulence in tomato leaves. These results suggested that retarded growth by overexpression of XvDMT2 might have contributed to the reduced virulence. In agreement with our observation, Banas et al. (2011) reported that DNA methylation plays a role in bacterial virulence by regulating the expression of various sugar transport genes in *Streptococcus*.

In addition, the comparative proteomic data revealed that abundance of 3-oxoacyl-ACP reductase (AOY69041), GTP diphosphokinase (AOY66666), GNAT family N-acetyltransferase (AOY66705), and phospholipase (AOY66479) was negatively affected by overexpression of XvDMT2, but not XvDMT1 (Supplementary Table 7). These proteins are known to be involved in the virulence of diverse pathogenic bacteria. It was reported that a 3-oxoacyl-ACP reductase deletion mutant of *X. campestris* pv. *campestris* attenuated virulence in cabbages (Yu et al., 2019). GTP diphosphokinase is required for (p)ppGpp production and virulence in animal pathogens (Dean et al., 2009; Dasgupta et al., 2019). GNAT family N-acetyltransferase is associated with producing virulence factors (Greene et al., 2015). Phospholipases are one of the well-characterized virulence factors (Flores-Diaz et al., 2016). Therefore, it is postulated that the low abundance of virulence-related proteins is possibly responsible for the reduction of virulence in *Xe*(XvDMT2).

Biofilm formation was reduced in *Xe*(XvDMT2) (Figure 4). Alternatively, *Xe*(XvDMT2) was less adhesive compared with *Xe*(EV) in the given condition. Biofilm formation is a critical factor for bacterial virulence, and it is also recognized that adhesion in bacteria is crucial for biofilm formation (Arciola et al., 2018). Besides, it cannot be ruled out that the reduced biofilm formation in *Xe*(XvDMT2) may be due to the retarded growth in the micro-anaerobic condition at the bottom of the 96-well plate. In animal pathogens, biofilm formation has been reported to critically determine bacterial virulence by promoting microbial colonization (Di Domenico et al., 2017). Mutant strains of *Xanthomonas* spp., showing reduced biofilm formation, were shown to be less virulent than the wild-type strain (Park and Han, 2017; Bae et al., 2018a,b). In addition to biofilm formation, EPS production has also been indispensable for virulence in *Xanthomonas* spp. (Kamoun and Kado, 1990; He et al., 2010). *Xe*(XvDMT2) also showed the reduced EPS production. Overexpression of EadM in *Xag* led to the reduction in the production of EPS and its virulence (Park et al., 2019). Thus, it could be speculated that the reductions in biofilm formation and EPS production were responsible for the reduced virulence of *Xe*(XvDMT2). We identified several diverse cell wall/membrane-related proteins in our performed comparative proteomic analysis. For instance, YajC, a preprotein translocase, was shown to be negatively regulated by XvDMT1 and XvDMT2 (Supplementary Table 6). Briefly, YajC is involved in the tolerance of *Lactobacillus buchneri* and *E. coli* to EtOH by binding to the SecD/SecF complex (Liu et al., 2016). In agreement with the above observation, the tolerance to EtOH and sorbitol was increased in *Xe*(XvDMT1), but not in *Xe*(XvDMT2) in our study (Figure 5A). Unexpectedly, tolerance to polymyxin B was significantly reduced in *Xe*(XvDMT1), but not *Xe*(XvDMT2). These data suggested that XvDMT1, rather than XvDMT2, might be associated with the tolerance against stress conditions.

Finally, the putative methylation motif of XvDMT1 was successfully assigned using SMRT sequencing (Figure 6A). Based on our results, XvDMT1 likely transfers a methyl group to the second adenine on the TACGAG motif, creating TACGmAG, which is a previously uncharacterized methylation motif. Our results demonstrated that XvDMT1 specifically recognized the TACGAG sequence and is a 6mA DNA MTase. Most bacterial DNA MTases recognize palindromic motifs, such as GATC (Dam) and CC(A/T)GG (Dcm); however, TACGAG is not palindromic. Therefore, we postulate that XvDMT1 might produce hemimethylated sites on double-stranded DNA in *Xe*, possibly representing a new class of DNA MTases. Using four methylation-sensitive restriction enzymes, we demonstrated that XvDMT2 is a site-specific, 5mC DNA MTase (Figure 6B). The *Bsp*119I fragmentation patterns did not differ among *Xe*(EV), *Xe*(XvDMT2), and *Xe*(XvDMT1). Based on the *Msp*JI, McrBC, and *Eco*RII cleavage patterns, we postulated that XvDMT2 might recognize the 5′-(A/C/G)VC(A/T)G(A/C/G)-3′ palindromic motif and methylate the cytosine. Although we tried to purify XvDMT proteins using diverse expression systems in *E. coli*, we were unsuccessful. Therefore, we are unable to show the direct methylation activity of XvDMTs, and hard to predict how overexpression of XvDMT2 reduces virulence in *Xe* at this point.

In this study, we identified that overexpression of XvDMT1 and XvDMT2 displayed pleiotropic effects in diverse phenotypes, including virulence. XvDMT1 and XvDMT2 might play distinct roles in various biological mechanisms, although some processes might be affected by both proteins. However, we could not elucidate the molecular functions of both proteins. Although we had used the marker exchange mutagenesis and in-frame deletion over a year to characterize the roles of XvDMT1 and XvDMT2 at the molecular level, the knockout mutants were not generated. We could speculate that XvDMT1 and XvDMT2 are possibly associated with growth in sugar sources, biofilm formation, and EPS production, which may have effects on *Xe* virulence, through proteomic analysis and phenotypic observation. Finally, we predicted putative methylation motifs recognized by XvDMT1 and XvDMT2, which were previously uncharacterized DNA MTases. This study sheds light on the biological functions of DNA MTases in prokaryotes.

## Data Availability Statement

The datasets presented in this study can be found in online repositories. The names of the repository/repositories and accession number(s) can be found in the article/Supplementary Material.

## Author Contributions

S-WH conceived the study. S-WH and H-JP designed the experiments. H-JP, JL, and LH conducted the experiments. HS and WS conducted the methylome analysis. H-JP and S-WH analyzed the data and prepared the manuscript. All authors reviewed the manuscript.

## Conflict of Interest

H-JP was employed by the company Seoul Clinical Laboratories. The remaining authors declare that the research was conducted in the absence of any commercial or financial relationships that could be construed as a potential conflict of interest.

## Footnotes

^1^See www.ebi.ac.uk/pride/archive/projects/PXD019554
