# Supplementary material for: Two DNA Methyltransferases for Site-Specific 6mA and 5mC DNA Modification in Xanthomonas euvesicatoria
Source: Front Plant Sci. 2021 Mar 24;12:621466. doi: 10.3389/fpls.2021.621466 (PMC8025778; doi:10.3389/fpls.2021.621466)
Supplement: Supplementary file 1 [file Table_1.DOCX]

Supplementary Table 1. Bacterial strains and plasmids in this study.

| Strains and plasmid | Characteristics | Source or reference |
| --- | --- | --- |
| *Escherichia* *coli* | |  |
| DH5α | F-, `80*dlaczZ*Δ*M15*, Δ(*lacZY*A‐argF), U169, *deoR*, RecA1, *end*A1, *hsd*R17, gyrA96, *thit*‐1, relA1, *supE44* | Promega |
| ER3413 | DNA methyltransferases deficient strain | Brian P et al.,  2015 |
| ER3413(EV) | ER3413 carrying pBBR1-MCS5, Gm^r^ | This study |
| ER3413(XvDMT1) | ER3413 carrying pMCS5-XvDMT1, Gm^r^ | This study |
| ER3413(XvDMT2) | ER3413 carrying pMCS5-XvDMT2, Gm^r^ | This study |
| *Xanthomonas* *euvesicatoria* | |  |
| 85-10 | Wilde type, pepper race 2,Rif^r^ | Canteros B, 1990 |
| *Xe*(EV) | *Xe* carrying pBBR1-MCS5, Gm^r^ | This study |
| *Xe*(XvDMT1) | *Xe* carrying MCS5-XvDMT1, Gm^r^ | This study |
| *Xe*(XvDMT2) | *Xe* carrying MCS5-XvDMT2, Gm^r^ | This study |
| Plasmids | |  |
| pGem T-easy | TA cloning vector, Am^r^ | Promega |
| pGem T-XvDMT1 | pGem T-easy carrying a 2810-bp XvDMT1 fragment containing 6×His-tag at C-terminus, Am^r^ | This study |
| pGem T-XvDMT2 | pGem T-easy carrying a 1646-bp XvDMT2 fragment containing 6×His-tag at C-terminus, Am^r^ | This study |
| pBBR1-MCS5 | Broad-host-range vector, lac promoter, Gm^r^ | Kovach et al.,  1995 |
| pMCS5-XvDMT1 | pBBR1-MCS5 carrying a 2810-bp with 6×His-tag at N-terminus from pGem T-XvDMT1, Gm^r^ | This study |
| pMCS5-XvDMT2 | pBBR1-MCS5 carrying a 1646-bp with 6×His-tag at N-terminus from pGem T-XvDMT2, Gm^r^ | This study |

Rif^r^, Gm^r^, and Am^r^ represent resistance to rifarmpicin, gentamycin and ampicillin.
