# Supplementary material for: Two DNA Methyltransferases for Site-Specific 6mA and 5mC DNA Modification in Xanthomonas euvesicatoria
Source: Front Plant Sci. 2021 Mar 24;12:621466. doi: 10.3389/fpls.2021.621466 (PMC8025778; doi:10.3389/fpls.2021.621466)
Supplement: Supplementary file 2 [file Table_2.DOCX]

Supplementary Table 2. Proteins and peptide spectral matches (PSM) between *Xe*(EV), *Xe*(XvDMT1), and *Xe*(XvDMT2) in liquid chromatography-tandem mass spectrometry data.

| Strains | 1st | | 2nd | | | | 3rd | | shared proteins in 3 biological replicates |
| --- | --- | --- | --- | --- | --- | --- | --- | --- | --- |
|  | protein | PSM | | protein | PSM | protein | | PSM |  |
| *Xe*(EV) | 1110 | 56603 | | 1027 | 56601 | 1132 | | 56590 | 993 |
| *Xe*(XvDMT1) | 1162 | 66084 | | 1156 | 63420 | 1171 | | 64494 | 1109 |
| *Xe*(XvDMT2) | 1204 | 68869 | | 1181 | 65033 | 1199 | | 66933 | 1144 |
