# Supplementary material for: Two DNA Methyltransferases for Site-Specific 6mA and 5mC DNA Modification in Xanthomonas euvesicatoria
Source: Front Plant Sci. 2021 Mar 24;12:621466. doi: 10.3389/fpls.2021.621466 (PMC8025778; doi:10.3389/fpls.2021.621466)
Supplement: Supplementary file 10 [file Data_Sheet_1.PDF]

A

```
A1AQ_RS0115075      MNAVEIEQAITDLAEQPFDRAEFPYAFLEAFGNKATTIKRLRAGASNKSDLGGVLQTSNI      60
CBW57_RS00470       MNAVEIEQAITDLADQPFDPIEFPYAFLEAFGNKATTIKRLRAGASNKSDLGGVLQTNNI      60
XvDMT1              MNAVEIEQAITDLAEQPFDGAEFPYAFLEAFGNKATTIKRLRAGASNKSDLGGVLQTSNI      60
CGU41_31265         MNAVEIEQAITDLAEQSFDSAEFPYAFLEAFGNKETTIKRLRAGASNKSDLGGVLQTSNI      60
                    **************:* **  ************* *********************.**

A1AQ_RS0115075      HILTCEVGRVTQTLAALKASPATVKAKAKFILATDGADFEAEDLTSGETVACAFKDFPDH      120
CBW57_RS00470       HILVSEAGLVTQMLTALKASTATGKAKAKFILATDGEDFEAEDLTSGQTVACAFKDFPDH      120
XvDMT1              HILTCDAGRVTQTLAALKASPATAKAKAKFILATDGADFEAEDLTSGETVACAFKDFPDH      120
CGU41_31265         HILTCNAGQVTTALKALKASPATAKAKARFILATDGVDFEAEDLTSGLTVACAFKDFPDH      120
                    ***..:.* **  * ***** ** ****:******* ********** ************

A1AQ_RS0115075      FGFFLLLAGISTVRQISENAFDIRATSRLNRLYVELLKDNPEWGTAERRHDMNNLMARLI      180
CBW57_RS00470       FGFFLPLAGISTVRQISENSFDIRATSRLNRLYVELLKDNPEWGTARRRHDMNQFMSRLI      180
XvDMT1              FGFFLPLAGISTVRQISENAFDIRATSRLNRLYVELLKDNPEWGTAERRHDMNNLMARLI      180
CGU41_31265         FGFFLPLAGISTVRQISENAFDIRATSRLNRLYVELLKDNPEWGTAERRHDMNKLMARLI      180
                    ***** *************:**************************.******::*:***

A1AQ_RS0115075      FCFFAEDTDIFIGKGRFTETVAQMSAKDSSNTHEVIATLFRTMNTKREDRAAAKIPRWAE      240
CBW57_RS00470       FCFFAEDTDIFIGKGCFTNTIAQMSERDASNMHDVIAMLFLAMNTPRDERANVGIPRWAA      240
XvDMT1              FCFFAEDTDIFVGKGRFTETVAQMSAKDSSNTHEVIATLFRAMNTKREDRAAAKVPRWAE      240
CGU41_31265         FCFFAEDTDIFVGKGRFTETVAQMSAKDSSNTHEVLATLFRAMNTKREDRAAAKIPRWAE      240
                    ***********:*** **:*:**** :*:** *:*:* ** :*** *::** . :****

A1AQ_RS0115075      DFPYVNGQLFSGGDEVPRFSKIARSYLLHVGGLDWTKINPDIFGSMIQAVAEDEERGELG      300
CBW57_RS00470       EFPYVNGALFSGSGEVPRFSRIARSYLLHVGNLDWTRINPDIFGSMIQAIAEDEERGELG      300
XvDMT1              DFPYVNGQLFSGGDEVPRFSKIARSYLLHVGGLDWTKINPDIFGSMIQAVAEDEERGELG      300
CGU41_31265         DFPYVNGQLFSGGDEVPRFSKIARSYLLHVGSLDWTKINPDIFGSMIQAVAEDEERGELG      300
                    :****** ****..******:**********.****:************:**********

A1AQ_RS0115075      MHYTSVPNILKVLNPLFLDDLREKLDEAGDNARMLLNLRKRIAKIRVFDPACGSGNFLVI      360
CBW57_RS00470       MHYTSVPNILKVLNPLLLDELRAKLEDASDNPRTLLNLRKRMARIRVFDPACGSGNFLVI      360
XvDMT1              MHYTSVPNILKVLNPLFLDDLRSKLEEAGDNPRTLLNLRKRIAKIRVFDPACGSGNFLVI      360
CGU41_31265         MHYTSVPNILKVLNPLFLDDLRAKLEEAGDNARMLLNLRKRIAKIRVFDPACGSGNFLVI      360
                    ****************:**:** **::*.** * *******:*:****************

A1AQ_RS0115075      AYKEMRAIEAEINRRRGEPDRASEIPLTNFRGIELRDFPAEIARLALVIAEYQCDVLYRG      420
CBW57_RS00470       AYKEMRAIEAEINQRRDEPDCASEIPLTNFRGIELRDFPAEIARLALIIAEYQCDVLYRG      420
XvDMT1              AYKEMRAIEAEINKRRGEPDRASGIPLTNFRGIELRDFPAEIARLALVIAEYQCDVLYRG      420
CGU41_31265         AYKEMRAIEAEINKRRGEPDRASDIPLTNFRGIELRDFPAEIARLALVIAEYQCDVLYRG      420
                    *************:**.*** ** ***********************:************

A1AQ_RS0115075      QKLALAEFLPLRNENWITCGNALRLDWLSICPSTGIGVKVQ-ADDLFETPLDQAEIDFEN      479
CBW57_RS00470       QKLALADFLPLRYENWITCGNALQLDWLSICPPSGTSVKVQ-ANSLFGFPLDQAEIDFEN      479
XvDMT1              QKLALAEFLPLRNENWITCGNALRLDWLSICPPTGTGVKVQ-ADDLFDSPLDQAEIDFEN      479
CGU41_31265         QKLALAEFLPLRNENWITCGNALRLDWLSICPPTGTGVKVRGADDLFGSPLDQAEIAFEN      480
                    ******:***** **********:******** :* .***: *:.**  ******* ***

A1AQ_RS0115075      EGGETYICGNPPYSGKGKKEAQHLADMDHIFTSRTDKYGYVDFVGCWFVLAADYCRKADA      539
CBW57_RS00470       EGGETYICGNPPYLGSRDQKEEQKDDLRVLFDKRIENWKSLDYVAGWWIKATDYCTYTDA      539
XvDMT1              EGGETYICGNPPYLGSRDQKEEQKADLRLLFDKRVENWKSLDYVAGWWIKAADYCTHTEA      539
CGU41_31265         EGGETYICGNPPYLGSRDQKDEQKADLQLLFDKRVGNWKSLDYVAGWLMKATDYCTNTRS      540
                    ************* *. .:: ::  *:  :* .*  ::  :*:*. * : *:***  : :

A1AQ_RS0115075      IAALVATNSVCQGRQLPQLWPHVLGNDIEIVFAHPSFKWANNAARNAGVTCVIIGIARKG      599
CBW57_RS00470       IASFVSTNSICQGLQVPVLWPAIFASGCQIDFAYTSFRWANLASHNAGVTVVIVGITTKP      599
XvDMT1              IAAFVSTNSICQGLQVPVLWPAIFASGCQIDFAYTSFRWANLASHNAGVTVAIVGITTQP      599
CGU41_31265         IAAFVTTNSICQGLQVPVLWPAIFDRGCQIEFAHTSFRWANLASHNAGVSVAIVGITTQP      600
                    **::*:***:*** *:* *** ::  . :* **: **:**:*:*** *::****: .*:**: :

A1AQ_RS0115075      ARSEKVLYEDD----FARTVTNINAYLISGSDIVVESIPRPLSPSLPAMQTGSVPNDGGN      655
CBW57_RS00470       RSPRRLFSLDSSGNTIKHQGSYINAYLALGETVIVEKASQPLST-LNEMTFGNTPIDGGL      658
XvDMT1              RSPRRLFSLDSSGSTIERQGSHINAYLALGETVIVEKASQPLSA-LSEMTFGNTPIDGGL      658
CGU41_31265         RSPRRLFSLDSSGNTIERQGSHINAYLALGESVIVEKASQPLSA-ISEMTFGNTPIDGGL      659
                      .:::  *.     : :  : *****  *. ::**.  :***  :  *  *..* ***

A1AQ_RS0115075      LILDPNEYRELALQAPHTVRLTHRFFGSADYLQGDVRYCLVIPDDEANEAALTPNIGERL      715
CBW57_RS00470       LLLSRDERDALQLTPEQQSRFVRRIVGSAEFIRGLERYCLWIEDQHLAEAQTILPLAQRI      718
XvDMT1              LLLSRDERDALQLTSEQQRRFIRRIVGSAEFIRGLERYCLWIEDQHLAEAQAILPLAQRI      718
CGU41_31265         LLLSRDERDALQLTPGQLCRFVRRIVGSAEFIRGLERYCLWIEDQHLAEAQAIPPLAQRI      719
                    *:*. :*   * *   :  *: :*:.***::::*  **** * *:.  **      :.:*:

A1AQ_RS0115075      KRIAALRAVSKKKETREQLAKVPHRFQHRGDIPRRHAIIVPSVSSERREWFPTGILPAGT      775
CBW57_RS00470       RRVRDLRLNGGKT--ARDIAERSHQFQ-RMFIGKNSTLVVPSVSSESREYLPCGYEPAGT      775
XvDMT1              RGVRDLRLNGGKT--ARDIAERSHQFQ-RMFIGKNSTLVVPSVSSESREYLPCGYEPAGT      775
CGU41_31265         CGVRNLRLNGGKT--ARDIAERAHQFQ-RMFIGKYATLVVPSVSSESREYLPCGYEPAGT      776
                      :  **  . *.   .::*:  *:** *  * :  :::******* **::* *  ****

A1AQ_RS0115075      VISNLAFMILDAPQWNMALIASRLHLVWIATVCGKLETRYRYSNTLGWNTFPVPTLTEKM      835
CBW57_RS00470       VISNLAFALYDAPLWNMGLVASRLHLVWIATVCGKLETRYRYSNTLGWNTFPVPTLTEKN      835
XvDMT1              VVSNLAFALYDAPLWNMALIASRLHLVWIASVCGKMKTDFRYSNTLGWNTFPVPTLTEKN      835
CGU41_31265         VVSNLAFALYDAPLWNMALIASRLHLIWIATVCGKLETRYRYSNTLGWNTFPVPTLTEKN      836
                    *:***** : *** ***.*:******:***:****::* :******************

A1AQ_RS0115075      KADLTRCAEDILLAREHHFPASIADLYDPENMPADLRAAHDRNDEVLERIYIGRRFKNDT      895
CBW57_RS00470       KADLTRCAEDILLAREHHFPATIADLYDPEKMPADLRTAHDRNDEVLERIYIGRRFKNDT      895
XvDMT1              KADLTRCAEEILLAREHHFPATIADLYAPDGMPADLRTAHDRNDEVLERIYIGRRFKNDT      895
CGU41_31265         KADLTRCAEEILLAREHHFPATIADLYAPENMPADLRAAHDRNDEVLERIYIGRRFKNDT      896
                    *********:***********:***** *: ******:**********************

A1AQ_RS0115075      ERLEKLFDLYTKMTASAAPAKGKKRKAGANA     926
CBW57_RS00470       ERLEKLFDLYTKMIASVAPAKGKKRKTGANE     926
XvDMT1              ERLEKLFDLYTKMTASVAPAKGKKRKVGANA     926
CGU41_31265         ERLEKLFDLYTKMTASAALVTGKKRKAGANA     927
                    ************* **.* ..*****.***
```

B

```
Dcm_X.citri          MHPQ--------PCAPLLYGSVCSGIEAVSLAWQPLGLEAAWFAEIEPFPSAVLAHHYPH     52
Dcm_P.aeruginosa     MNPQPRTPRGAPQAAPLLYGSVCSGIEAVSLAWQTLGLEAAWFAEIEPFPSAVLAHRYPR     60
XvDMT2               MNPQPRTPRGAPQAAPLLYGSVCSGIEAVSLAWQPIGLEAAWFAEIEPFPSAVLAHRYPR     60
XF_1774              MNPQPRTPRGAPQAAPLLYGSVCSGIEAVSLAWQPLGLEAAWFAEIEPFPSAVLAHRYPR     60
                     *:**         .******************** :********************:**:

Dcm_X.citri          VPNLGDMTTIARQVHAGTVPAPDILVGGTPCQSFSVAGARRGLDDPRGALTLAYVELANA     112
Dcm_P.aeruginosa     VPNLGDMTAIARQVRAGTVPAPDILVGGTPCQSFSVAGSRRGLDDPRGALSLAYVELANA     120
XvDMT2               VPNLGDMTAIARQVRAGTVPAPDILVGGTPCQSFSVAGSRRGLDDPRGALSLAYVELANA     120
XF_1774              VPNLGDMTAIARQVRAGTVPAPDILVGGTPCQSFSVAGSRRGLDDPRGALSLAY-ELANA     119
                     ********:*****:***********************:***********:*** *****

Dcm_X.citri          IDQARHQNDRSPATLVWENVPGVLSDRSNAFGNFLGALAGESRALQPPGEKWAHAGYVSG     172
Dcm_P.aeruginosa     IDQIRHQDRRSPATLVWENVPGVLNDRCNAFGQFLAALAGESRALQPPGGRWAHAGCVSG     180
XvDMT2               IDQIRHQDRRPAATLVWENVPGVLNDRCNAFGQFLAALAGESRALQPPGGRWAHAGCVSG     180
XF_1774              IDQIRHQDRRSPATLVWENVPGVLNDRSNAFGHFLGALAGESRALQPPGDRWAHAGCVSG     179
                     *** ***: *  ************.**.****:**.************* :***** ***

Dcm_X.citri          PRRRIAWRVLDAQYFGVAQRRKRVFLVASGGDGFDPVEVLFERTGLRGDSSAGSAPWQEA     232
Dcm_P.aeruginosa     PRRRIAWRVLDAQYFGVAQRRKRVFLVASGGNDLDPAEVLFERAGLLGDSCAGRAPWQEA     240
XvDMT2               PRRRIAWRVLDAQYFGVAQRRKRVFLVASGGNDLDPAEVLFERAGLLGDSCAGRAPWQEA     240
XF_1774              PRRRIAWRVLDAQYFGVAQRRKRVFLVASGGDDLDPAEVLFERAGLLGDSCAGRAPWQEA     239
                     *******************************:.:**.******:** ***.** ******

Dcm_X.citri          ARAAGPSAEAAG------GYAGLKSSYGEVKTTFGFSGGIGPVDVAACLMAAGPKHDIRT     286
Dcm_P.aeruginosa     ARDAGPGAEAAG---GYAGFAGLKQPYGKVTTTFGFSGGIGPVDVAACLMAAGPKHDIRT     297
XvDMT2               AHDAGPGAEAAGACAGLAGFAGLKQPYGKITTTFGFSGGIGPVDVAACLMAAGPKHDIRT     300
XF_1774              AHDAGPGAEAAGACAGLAGFAGLKQPYGKVTTTFGFSGGIGPVDVAACLMAAGPKHDIRT     299
                     *: ***.*****      *:****. **::.*****************************

Dcm_X.citri          ETFMVQSVAGSITHTLDTANGGKGSSEDGTGKGVPIIAFTAQGSGADATMDLTPTLRAGG     346
Dcm_P.aeruginosa     ETFMVQSVAGSIAHTLDTANNGKGSSEDGTGKGVPIIAFTAQGSGADATLDLTPTLRAGG     357
XvDMT2               ETFMVQSVAGSIAHTLDTANNGKGSSEDGTGKGVPIIAFTAQGSGADATLDLTPTLRAGG     360
XF_1774              ETFMVQSVAGSIAHTLDTANNGKGSSEDGTGKGVPIIAFTAQGSGADATLDLTPTLRAGG     359
                     ************:*******.****************************:**********

Dcm_X.citri          HRSSHANAGVVPAIAFAQNHRCEVRFESGHGQVACTVLSNGKPGYGVPMIACVALHDRQN     406
Dcm_P.aeruginosa     HRNSHANAGVVPAIAFAQNNRGEVRFESGHGQVACTVLSNGKPGYGVPMVACVSLRGREQ     417
XvDMT2               HRNSHANAGVVPAIAFAQNNRGEVRFESGHGQVACTVLSNGKPGYGVPMVACVSLRGREQ     420
XF_1774              HRNSHANAGVVPAIAFAQNNRGEVRFESGHGQVACTVLSNGKPGYGVPMVACVSLRGREQ     419
                     **.****************:* ***************************:***:*:.*::

Dcm_X.citri          GLAAELGSGAN------------GHVLAPDHEAHFRYDWNDPIPRDWSQWRVRRLMPVEC     454
Dcm_P.aeruginosa     GLAAELGGSVAGALRTSGGGADKPYVLAPDFEAHFRYDWNDPGPGDWSHWRVRRLMPVEC     477
XvDMT2               GLAAELGGTVAGALRTSGGGADKPYVLAPDFEAHFRYDWNDPGPGDWSHWRVRRLMPVEC     480
XF_1774              GLAAELGGTVAGALRISGGGADKPYVLAPDFEAHFRYDWNDPGPGDWSHWRVRRLMPVEC     479
                     *******. .              :*****.*********** * ***:***********

Dcm_X.citri          ERLQGMPDDYTLVPYRGKPAADAPRYKAIGNSMAMPCVAWLGQRLVQCLHKMGSIASD       512
Dcm_P.aeruginosa     ERLQGMPDDYTLITYRGKPAADAPRYKAIGNSMAVPCMAWLGQRLVHYLHKTGSTASD       535
XvDMT2               ERLQGMPDDYTLIPYRGKPAADAPRYKAIGNSMAVPCMAWLGQRLVQYLHKTGSTASD       538
XF_1774              ERLQGMPDDYTLIPYRGKPAADAPRYKAIGNSMAVPCMAWLGQRLVQYLHKTGSTASD       537
                     ************: ********************:**:********: *** ** ***
```

C

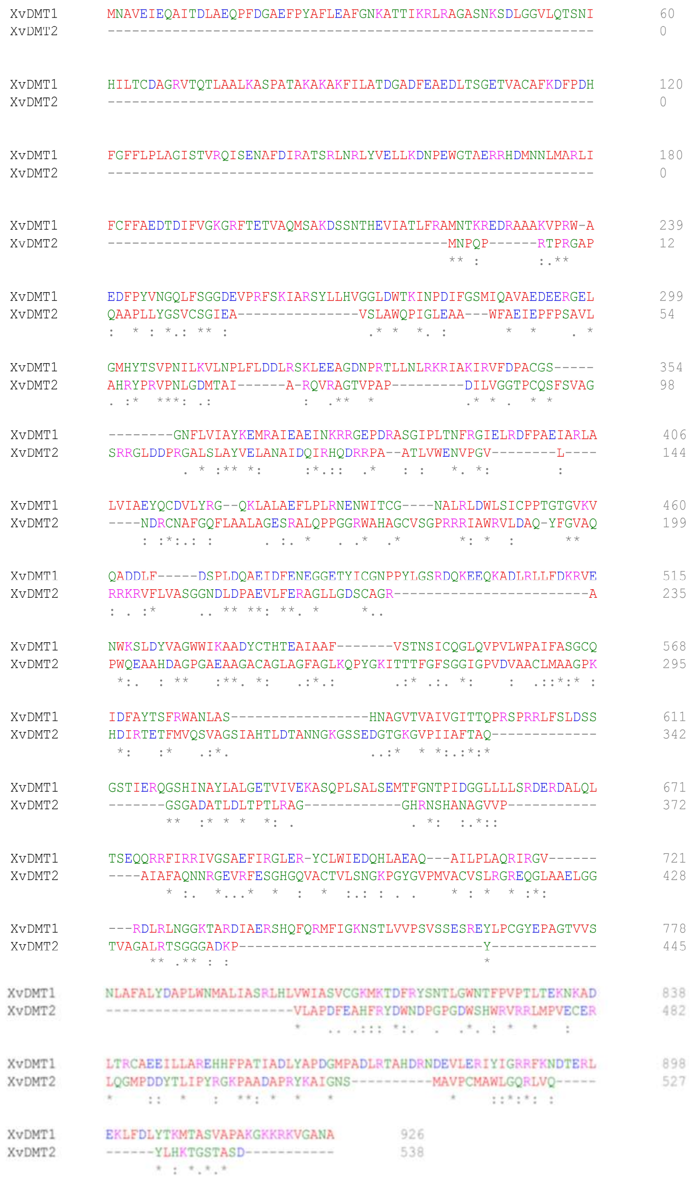

Supplementary Figure 1. Sequence alignment of XvDMT1, XvDMT2, and indicated homologs. The deduced amino acid sequences of (A) XvDMT1 and (B) XvDMT2 were compared with those of homologs in other bacteria using the CLUSTAL OMEGA program. (C) Sequence alignment of XvDMT1 and XvDMT2. A1AQ_RS0115075, CBW57_RS00470, and CGU41_31265 are putative site-specific DNA MTases in *Xanthomonas phaseoli*, *Yersinia intermedia*, and *Pseudomonas aeruginosa*, respectively. Dcm_*X. citri*, Dcm_*P. aeruginosa*, and XF_1771 are putative site-specific DNA methyltransferase in *Xanthomonas citri*, *P. aeruginosa,* and *Xylella fastidiosa*, respectively. "*", ":" and "." indicate identical residues, conserved substitutions, and semiconserved substitutions, respectively.
