# Supplementary material for: Two DNA Methyltransferases for Site-Specific 6mA and 5mC DNA Modification in Xanthomonas euvesicatoria
Source: Front Plant Sci. 2021 Mar 24;12:621466. doi: 10.3389/fpls.2021.621466 (PMC8025778; doi:10.3389/fpls.2021.621466)
Supplement: Supplementary file 11 [file Data_Sheet_2.pdf]

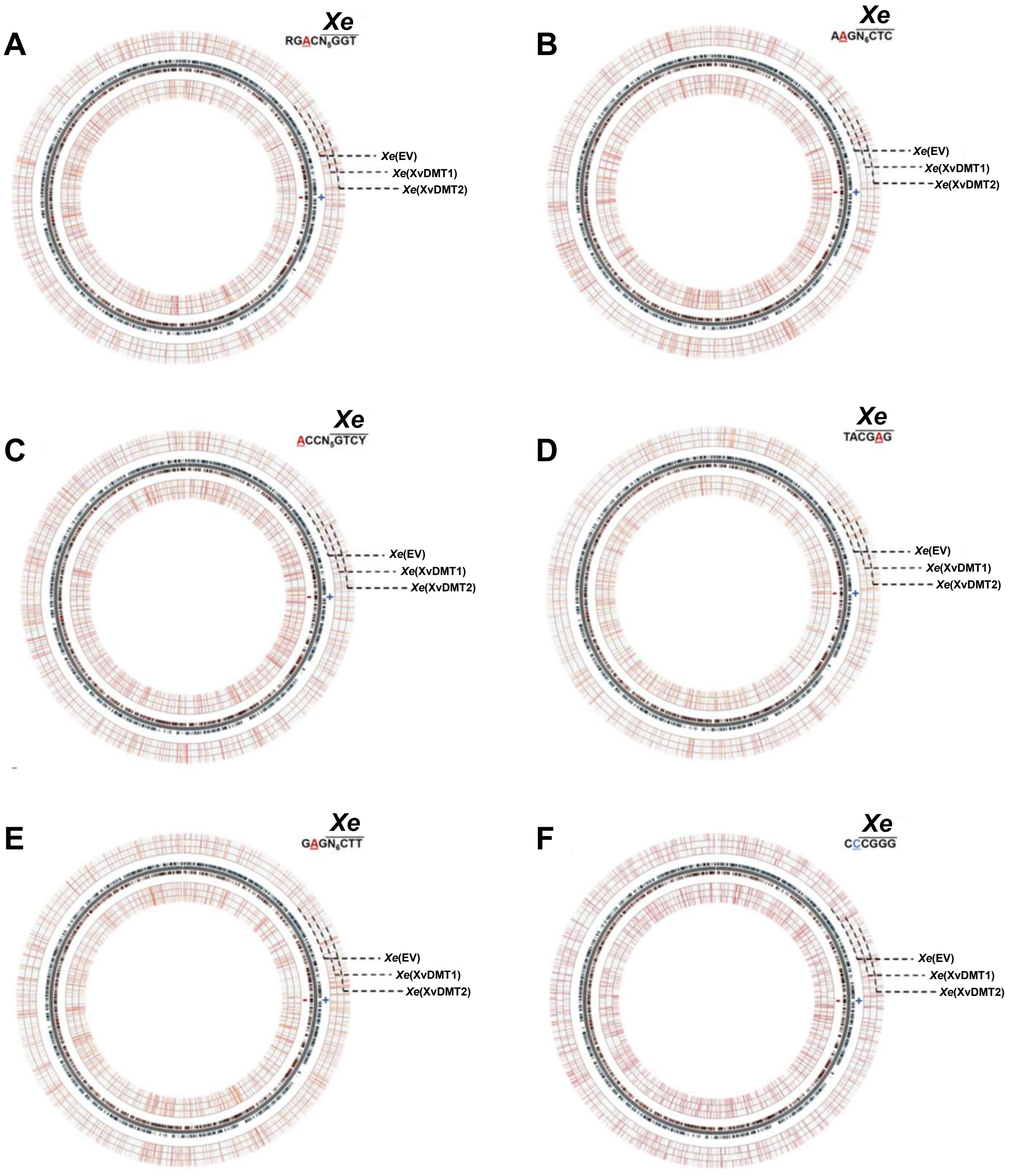

Supplementary Figure 2. Genome-wide methylation patterns in *Xe* strains. Methylation patterns of the six most frequently methylated motifs (A: RGACN$_5$GGT, B: AAGN6CTC, C: ACCN$_5$GTCY, D: TACGAG, E: GAGN$_6$CTT, and F: CCCGGG) were analyzed in *Xe*(EV), *Xe*(XvDMT1), and *Xe*(XvDMT2). Sites of all methylated motifs are shown in the genome Circos plot, with the height of the bars along with the methylated fractions of motifs.
