# Supplementary material for: Two DNA Methyltransferases for Site-Specific 6mA and 5mC DNA Modification in Xanthomonas euvesicatoria
Source: Front Plant Sci. 2021 Mar 24;12:621466. doi: 10.3389/fpls.2021.621466 (PMC8025778; doi:10.3389/fpls.2021.621466)
Supplement: Supplementary file 12 [file Data_Sheet_3.pdf]

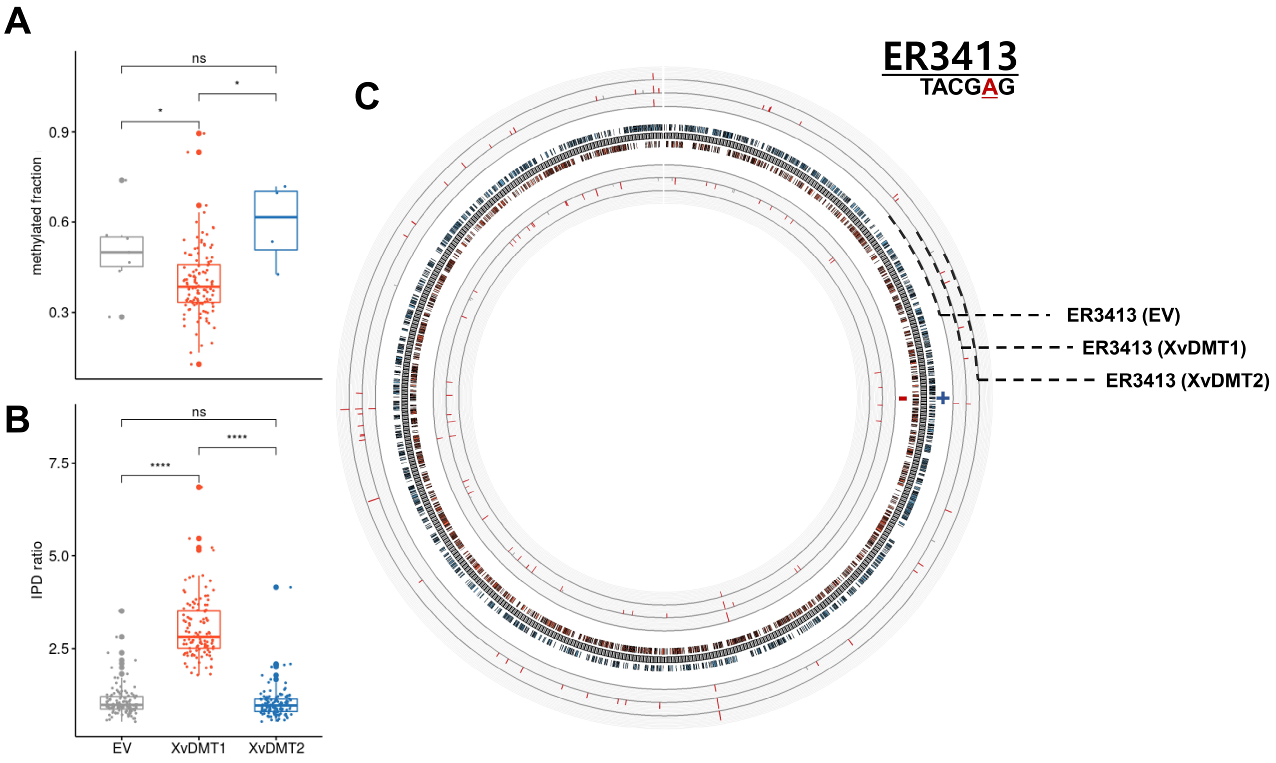

Supplementary Figure 3. Methylome analysis of a TACGAG motif using the ER3413 *E. coli* strain. (A) Methylated fraction of each specific motif for ER3413(EV), ER3413(XvDMT1), and ER3413(XvDMT2). Each position of the methylated motifs is plotted, showing the frequency of methylation. (B) Inter-pulse duration (IPD) values of a motif position in the genome. The IPD value is a pulse signal value during the incorporation of fluorescent nucleotides, indicating the position of the modification in DNA. (C) Genome-wide methylation pattern of a TACGAG motif in the ER3413 chromosome. Sites of all methylated motifs are shown in the genome Circos plot, with the height of the bars along with the methylated fractions of motifs being indicated by the same value as (A). *: $p \leq 0.05$, **: $p \leq 0.01$, ***: $p \leq 0.001$, ****: $p \leq 0.0001$.
