# Supplementary material for: Two DNA Methyltransferases for Site-Specific 6mA and 5mC DNA Modification in Xanthomonas euvesicatoria
Source: Front Plant Sci. 2021 Mar 24;12:621466. doi: 10.3389/fpls.2021.621466 (PMC8025778; doi:10.3389/fpls.2021.621466)
Supplement: Supplementary file 13 [file Data_Sheet_4.PDF]

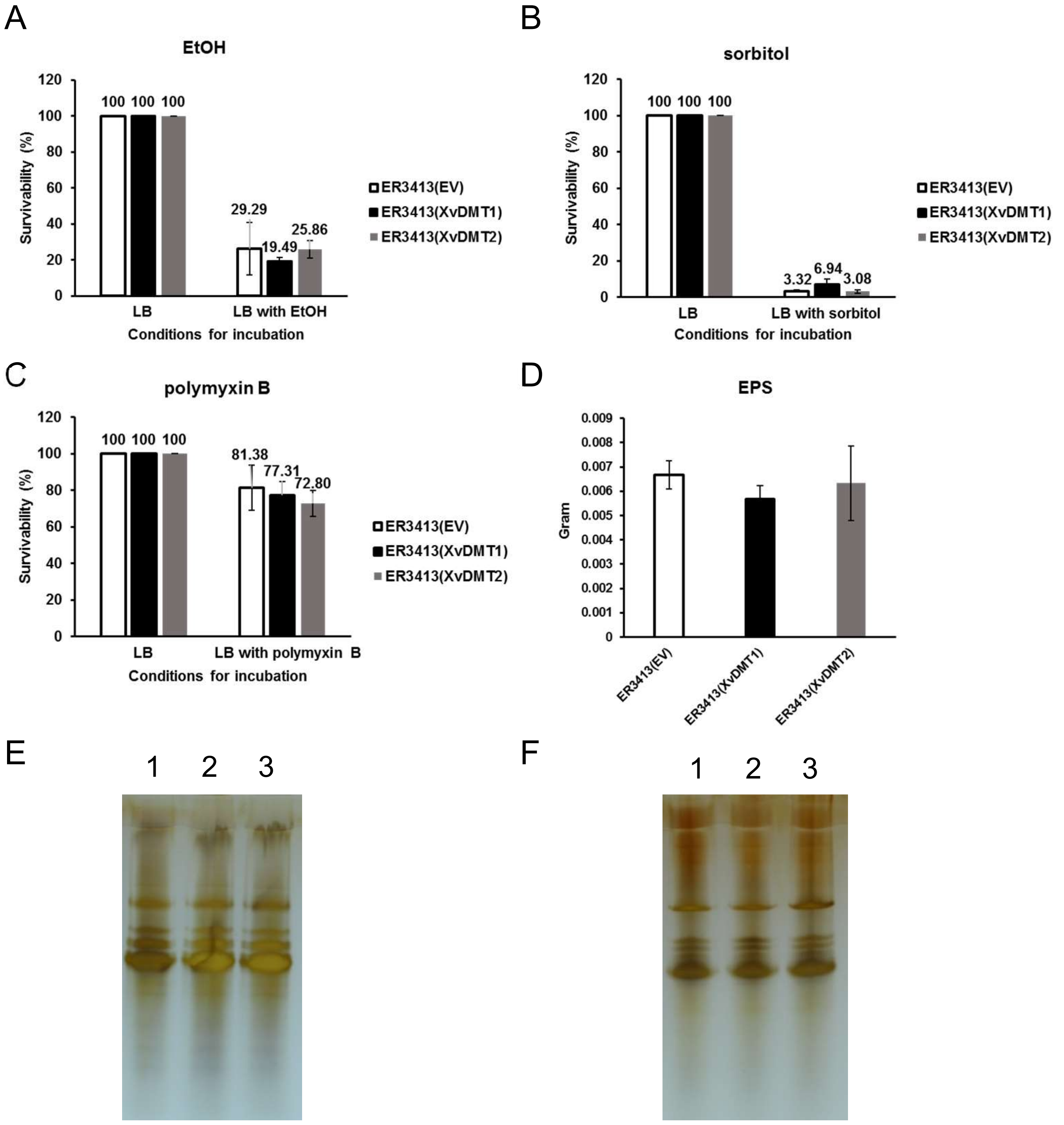

Supplementary Figure 4. Phenotype observations for *E. coli* strains and LPS profiles of *Xe* and *E. coli* strains. Tolerance to (A) EtOH, (B) sorbitol, (C) polymyxin B, and (D) production of EPSs in ER3413(EV), ER3413(XvDMT1), and ER3413(XvDMT2). There is no statistical difference among three strain. All experiments were carried out at least three times with three replicates. The (E) and (F) panels illustrate LPS profiles of *E. coli* and *Xe* strains, respectively. Here, 1, 2, and 3 indicate strains carrying an empty vector, XvDMT1, and XvDMT2, respectively.
